# Supplementary material for: Variability and performance of NHS England’s ‘reason to reside’ criteria in predicting hospital discharge in acute hospitals in England: a retrospective, observational cohort study
Source: BMJ Open. 2022 Dec 26;12(12):e065862. doi: 10.1136/bmjopen-2022-065862 (PMC9805825; doi:10.1136/bmjopen-2022-065862)
Supplement: Supplementary data [file bmjopen-2022-065862supp001.pdf]

Online Supplement: The variability and performance of NHS Englands' "Reason to Reside" criteria in predicting hospital discharge in acute hospitals in England

<u>Online Tables</u>

National Trusts	
•Airedale NHS Foundation Trust<br>•Ashford And St Peter's Hospitals NHS Foundation Trust<br>•Barking, Havering And Redbridge University Hospitals NHS Trust<br>•Barnsley Hospital NHS Foundation Trust<br>•Barts Health NHS Trust<br>•Bedfordshire Hospitals NHS Foundation Trust<br>•Blackpool Teaching Hospitals NHS Foundation Trust<br>•Bolton NHS Foundation Trust<br>•Bradford Teaching Hospitals NHS Foundation Trust<br>•Buckinghamshire Healthcare NHS Trust<br>•Calderdale And Huddersfield NHS Foundation Trust<br>•Cambridge University Hospitals NHS Foundation Trust<br>•Chelsea And Westminster Hospital NHS Foundation Trust<br>•Chesterfield Royal Hospital NHS Foundation Trust<br>•Countess Of Chester Hospital NHS Foundation Trust<br>•County Durham And Darlington NHS Foundation Trust<br>•Croydon Health Services NHS Trust<br>•Dartford And Gravesham NHS Trust<br>•Doncaster And Bassetlaw Teaching Hospitals NHS Foundation Trust<br>•Dorset County Hospital NHS Foundation Trust<br>•East And North Hertfordshire NHS Trust<br>•East Cheshire NHS Trust<br>•East Kent Hospitals University NHS Foundation Trust<br>•East Lancashire Hospitals NHS Trust<br>•East Suffolk And North Essex NHS Foundation Trust<br>•East Sussex Healthcare NHS Trust<br>•Epsom And St Helier University Hospitals NHS Trust<br>•Frimley Health NHS Foundation Trust<br>•Gateshead Health NHS Foundation Trust<br>•George Eliot Hospital NHS Trust<br>•Gloucestershire Hospitals NHS Foundation Trust<br>•Great Western Hospitals NHS Foundation Trust<br>•Guy's And St Thomas' NHS Foundation Trust<br>•Hampshire Hospitals NHS Foundation Trust<br>•Harrogate And District NHS Foundation Trust<br>•Homerton University Hospital NHS Foundation Trust<br>•Hull University Teaching Hospitals NHS Trust<br>•Imperial College Healthcare NHS Trust<br>•Isle Of Wight NHS Trust<br>•James Paget University Hospitals NHS Foundation Trust<br>•Kettering General Hospital NHS Foundation Trust<br>•King's College Hospital NHS Foundation Trust<br>•Kingston Hospital NHS Foundation Trust<br>•Lancashire Teaching Hospitals NHS Foundation Trust	•Salisbury NHS Foundation Trust<br>•Sandwell And West Birmingham Hospitals NHS Trust<br>•Sheffield Teaching Hospitals NHS Foundation Trust<br>•Sherwood Forest Hospitals NHS Foundation Trust<br>•Somerset NHS Foundation Trust<br>•South Tees Hospitals NHS Foundation Trust<br>•South Tyneside And Sunderland NHS Foundation Trust<br>•South Warwickshire NHS Foundation Trust<br>•Southport And Ormskirk Hospital NHS Trust<br>•St George's University Hospitals NHS Foundation Trust<br>•St Helens And Knowsley Teaching Hospitals NHS Trust<br>•Stockport NHS Foundation Trust<br>•Surrey And Sussex Healthcare NHS Trust<br>•Tameside And Glossop Integrated Care NHS Foundation Trust<br>•The Dudley Group NHS Foundation Trust<br>•The Hillingdon Hospitals NHS Foundation Trust<br>•The Newcastle Upon Tyne Hospitals NHS Foundation Trust<br>•South Warwickshire NHS Foundation Trust<br>•Southport And Ormskirk Hospital NHS Trust<br>•St George's University Hospitals NHS Foundation Trust<br>•St Helens And Knowsley Teaching Hospitals NHS Trust<br>•Stockport NHS Foundation Trust<br>•Surrey And Sussex Healthcare NHS Trust<br>•Tameside And Glossop Integrated Care NHS Foundation Trust<br>•The Dudley Group NHS Foundation Trust<br>•The Hillingdon Hospitals NHS Foundation Trust<br>•The Newcastle Upon Tyne Hospitals NHS Foundation Trust<br>•The Princess Alexandra Hospital NHS Trust<br>•The Queen Elizabeth Hospital, King's Lynn, NHS Foundation Trust<br>•The Rotherham NHS Foundation Trust<br>•The Royal Wolverhampton NHS Trust<br>•The Shrewsbury And Telford Hospital NHS Trust<br>•Torbay And South Devon NHS Foundation Trust<br>•United Lincolnshire Hospitals NHS Trust<br>•University College London Hospitals NHS Foundation Trust

•Leeds Teaching Hospitals NHS Trust<br>•Lewisham And Greenwich NHS Trust<br>•Liverpool University Hospitals NHS Foundation Trust<br>•London North West University Healthcare NHS Trust<br>•Maidstone And Tunbridge Wells NHS Trust<br>•Manchester University NHS Foundation Trust<br>•Medway NHS Foundation Trust<br>•Mid And South Essex NHS Foundation Trust<br>•Mid Cheshire Hospitals NHS Foundation Trust<br>•Mid Yorkshire Hospitals NHS Trust<br>•Milton Keynes University Hospital NHS Foundation Trust<br>•Norfolk And Norwich University Hospitals NHS Foundation Trust<br>•North Bristol NHS Trust<br>•North Cumbria Integrated Care NHS Foundation Trust<br>•North Middlesex University Hospital NHS Trust<br>•North Tees And Hartlepool NHS Foundation Trust<br>•North West Anglia NHS Foundation Trust<br>•Northampton General Hospital NHS Trust<br>•Northern Care Alliance NHS Foundation Trust<br>•Northern Devon Healthcare NHS Trust<br>•Northern Lincolnshire And Goole NHS Foundation Trust<br>•Northumbria Healthcare NHS Foundation Trust<br>•Nottingham University Hospitals NHS Trust<br>•Oxford University Hospitals NHS Foundation Trust<br>•Portsmouth Hospitals University National Health Service Trust<br>•Royal Berkshire NHS Foundation Trust<br>•Royal Cornwall Hospitals NHS Trust<br>•Royal Devon And Exeter NHS Foundation Trust<br>•Royal Free London NHS Foundation Trust<br>•Royal Surrey County Hospital NHS Foundation Trust<br>•Royal United Hospitals Bath NHS Foundation Trust	•University Hospital Southampton NHS Foundation Trust<br>•University Hospitals Birmingham NHS Foundation Trust<br>•University Hospitals Bristol And Weston NHS Foundation Trust<br>•University Hospitals Coventry And Warwickshire NHS Trust<br>•University Hospitals Dorset NHS Foundation Trust<br>•University Hospitals Of Derby And Burton NHS Foundation Trust<br>•University Hospitals Of Leicester NHS Trust<br>•University Hospitals Of Morecambe Bay NHS Foundation Trust<br>•University Hospitals Of North Midlands NHS Trust<br>•University Hospitals Plymouth NHS Trust<br>•University Hospitals Sussex NHS Foundation Trust<br>•Walsall Healthcare NHS Trust<br>•Warrington And Halton Teaching Hospitals NHS Foundation Trust<br>•West Hertfordshire Hospitals NHS Trust<br>•West Suffolk NHS Foundation Trust<br>•Whittington Health NHS Trust<br>•Wirral University Teaching Hospital NHS Foundation Trust<br>•Worcestershire Acute Hospitals NHS Trust<br>•Wrightington, Wigan And Leigh NHS Foundation Trust<br>•Wye Valley NHS Trust<br>•Yeovil District Hospital NHS Foundation Trust<br>•York And Scarborough Teaching Hospitals NHS Foundation Trust

Table S1. The names of the Hospital Trusts included in the national R2R reporting analysis
Legend. Data is presented anonymously in associated figures.

Procedure	OPCS Codes
Lower limb surgery within 48hrs	See Online supplement xls.
Thorax-abdominal-pelvic surgery with 72hrs	See Online supplement xls.
Invasive procedure within 24hrs	See Online supplement xls.

Table S2. Codes used to identify surgical interventions.
Legend. OPCS = OPCS Classification of Interventions and Procedures code used to identify the coded clinical entry.

	**2019**	**Remain**		
		Yes (+)	No (-)	Total
**eR2Ra**	Yes (+)	214,613	21,333	235,946
	No (-)	128,470	19,270	147,740
	Total	343,083	40,603	383,686
	**2020**	**Remain**		
		Yes (+)	No (-)	Total
**eR2Ra**	Yes (+)	177,852	18,283	196,135
	No (-)	97,119	17,606	114,725
	Total	274,971	35,889	310,860
	**2021**	**Remain**		
		Yes (+)	No (-)	Total
**eR2Ra**	Yes (+)	208,449	19,989	228,438
	No (-)	115,111	19,075	134,186
	Total	323,560	39,064	362,624

	**2019**	**Remain**		
		Yes (+)	No (-)	Total
**eR2Rab**	Yes (+)	301,018	30,176	331,194
	No (-)	42,065	10,427	52,492
	Total	343,083	40,603	383,686
	**2020**	**Remain**		
		Yes (+)	No (-)	Total
**eR2Rab**	Yes (+)	249,964	28,636	278,600
	No (-)	25,007	7,253	32,260
	Total	274,971	35,889	310,860
	**2021**	**Remain**		
		Yes (+)	No (-)	Total
**eR2Rab**	Yes (+)	291,576	30,847	322,423
	No (-)	31,984	8,217	40,201
	Total	323,560	39,064	362,624

Table S3a. Contingency tables showing the number of patients meeting criteria for eR2Ra and eR2ab and the corresponding number of patients who remain in hospital over the next 24 hours or do not (were discharged), for the in-patient population at 08.00.

	2019	Remain		
		Yes (+)	No (-)	Total
**eR2Ra**	Yes (+)	214,005	19,919	233,924
	No (-)	129,543	18,465	148,008
	Total	343,548	38,384	381,932
	**2020**	**Remain**		
		Yes (+)	No (-)	Total
**eR2Ra**	Yes (+)	178,709	17,343	196,052
	No (-)	98,123	17,692	115,815
	Total	276,832	35,035	311,867
	**2021**	**Remain**		
		Yes (+)	No (-)	Total
**eR2Ra**	Yes (+)	211,080	19,105	230,185
	No (-)	116,893	19,616	136,509
	Total	327,973	38,721	366,694

	2019	Remain		
		Yes (+)	No (-)	Total
**eR2Rab**	Yes (+)	299,551	28,334	327,885
	No (-)	43,997	10,050	54,047
	Total	343,548	38,384	381,932
	**2020**	**Remain**		
		Yes (+)	No (-)	Total
**eR2Rab**	Yes (+)	250,507	27,672	278,179
	No (-)	26,325	7,363	33,688
	Total	276,832	35,035	311,867
	**2021**	**Remain**		
		Yes (+)	No (-)	Total
**eR2Rab**	Yes (+)	294,260	30,038	324,298
	No (-)	33,713	8,683	42,396
	Total	327,973	38,721	366,694

Table S3b. Contingency tables showing the number of patients meeting criteria for eR2Ra and eR2ab and the corresponding number of patients who remain in hospital over the next 24 hours or do not (were discharged), for the in-patient population at 16.00.

	eR2Ra			eR2Rab		
Year	2019	2020	2021	2019	2020	2021
Sensitivity	0.63	0.65	0.65	0.88	0.91	0.90
Specificity	0.46	0.47	0.47	0.24	0.18	0.19
J statistic	9%	12%	12%	11%	9%	9%

Table S4. The Sensitivity, Specificity and J statistic calculations for Data presented in Table 5 of the main manuscript. A Contingency table showing the number of patients meeting criteria for eR2Ra and eR2ab

Population at 00:00	Not meeting eR2Rab criteria and discharged in subsequent 24 hours	Not meeting eR2Rab criteria and not discharged
n	22515	97326
**Age in years**: median (IQR)	60(45-74)	64(49-77)
**Sex (n, %)**	.	
Female	10833 (48.1%)	45345 (46.6%)
Male	11682 (51.9%)	51981 (53.4%)
**Self-reported ethnicity (n, %)**		
White	15761 (70.0%)	70194 (72.1%)
Mixed/ Multiple	411 (1.8%)	1549 (1.6%)
Asian/ Asian British	2952 (13.1%)	12194 (12.5%)
Black/ African/ Caribbean/ Black British	1274 (5.7%)	5857 (6.0%)
Other ethnic group	567 (2.5%)	2285 (2.3%)
Not known	1550 (6.9%)	5247 (5.4%)
**Co-morbidity count (n, %)**		
None	6544 (29.1%)	24873 (25.6%)
1-2	10321 (45.8%)	41401 (42.5%)
3 or more	5650 (25.1%)	31052 (31.9%)
**Morbidities (n, %)**		
Hypertension	9168 (40.7%)	43062 (44.2%)
Cerebrovascular disease	1512 (6.7%)	10128 (10.4%)
Atrial fibrillation	2947 (13.1%)	17096 (17.6%)
Ischaemic heart disease, angina, myocardial infarct	3810 (16.9%)	20962 (21.5%)
Diabetes (type 1 and 2)	4809 (21.4%)	24368 (25.0%)
Asthma	2644 (11.7%)	9899 (10.2%)
COPD	1594 (7.1%)	7255 (7.5%)
Interstitial Lung Disease	24 (0.1%)	129 (0.1%)
Chronic Kidney Disease	3135 (13.9%)	16633 (17.1%)
Any active Malignancy	3968 (17.6%)	17572 (18.1%)
Dementia (all types)	535 (2.4%)	3413 (3.5%)
**English Indices of deprivation**		
1	9448 (42.0%)	38534 (39.6%)
2	4638 (20.6%)	19841 (20.4%)
3	3888 (17.3%)	16630 (17.1%)
4	2200 (9.8%)	9432 (9.7%)
5	1644 (7.3%)	6914 (7.1%)
Missing	697 (3.1%)	5975 (6.1%)
Regained R2R criteria during stay? (n, %)	N/A	58609 (60.2%)
**Reason for regaining R2R criteria?**		
ICU		1727 (1.8%)
TAP surgery (72h)		263 (0.3%)
Lower limb surgery (24h)		95 (0.1%)
Invasive surgery (24h)	N/A	579 (0.6%)
Acute dependency level (48h)		7452 (7.7%)
NEWS >3 (24h)		20605 (21.2%)
O2 treatment (24h)		5111 (5.3%)

Intravenous fluids or treatments (24 hours, > tds)		27069 (27.8%)
GCS < or + 12 (24h)		183 (0.2%)
EOL care (24h)		165 (0.2%)
Increased dependency (48h)		10290 (10.6%)

Table S5. Demographics of patients not meeting R2R criteria on presentation to QEHB in the censor period. **Legend.** Data is number (percentage) of patients in a bed at 00:00 who either were or were not discharged in the subsequent twenty-four hours after eR2R assessment. Ethnicity was self-reported. Medical conditions were physician confirmed and checked against admission and linked primary care notes. English Indices of deprivation were calculated using postcode.

Online Supplement Figures

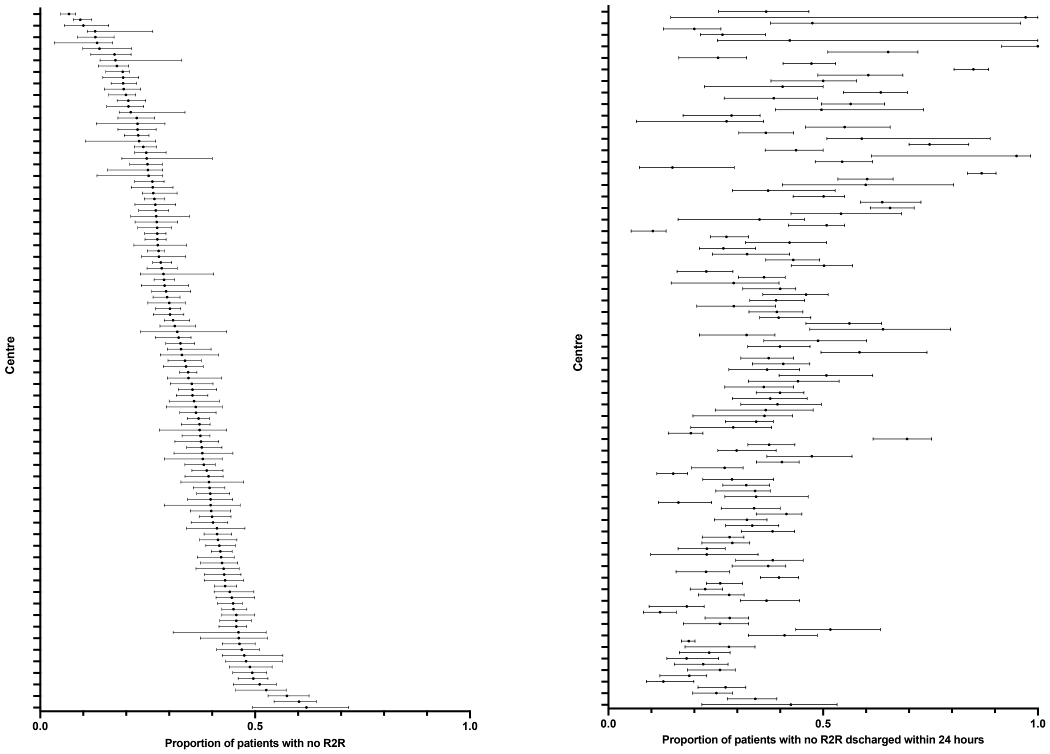

Figure S1. The proportions of patients with no right to reside (A), and proportions of these with no right to reside that were discharged within 24 hours (B). Analysis by week.

Legend: The proportions of patients not meeting the R2R, and the proportions of these patients that were discharged within 24 hours were extracted from daily reports for each national NHS centre. Data is presented as the median and IQR for the reporting period. We have ordered centres in both Figure 1 a and 1 b according to the median value of proportion of patients with R2R.

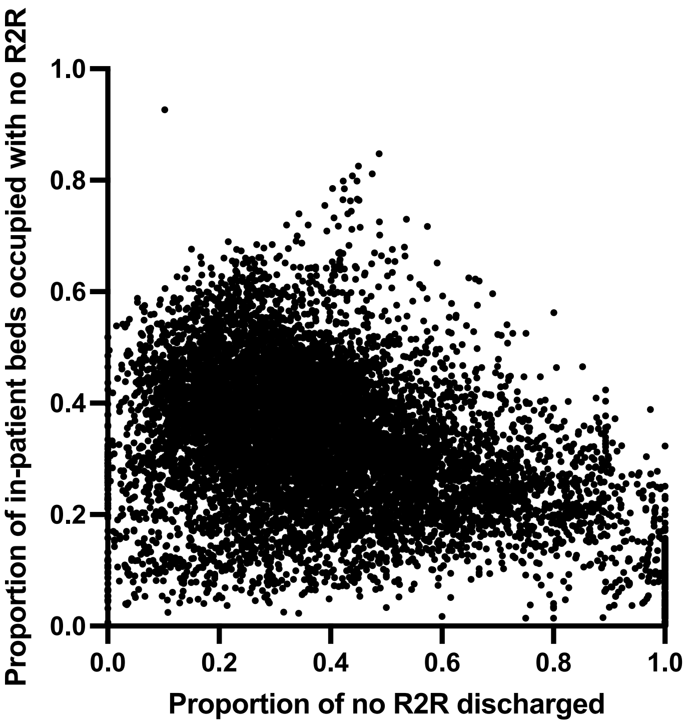

Figure S2. The proportions of patients with no R2R and of that group the proportion discharged over the next 24 hours

Legend: The proportions of patients not meeting the R2R, and of that group the proportion of patients discharged within 24 hours, reported to SDCS from 29 Nov 2021 – 20 Feb 2022 across 121 centres. Each dot represents result for a single centre-day. The two metrics were associated (slope = -0.21, $p<0.0001$) but the correlation was low (R^2=0.12).

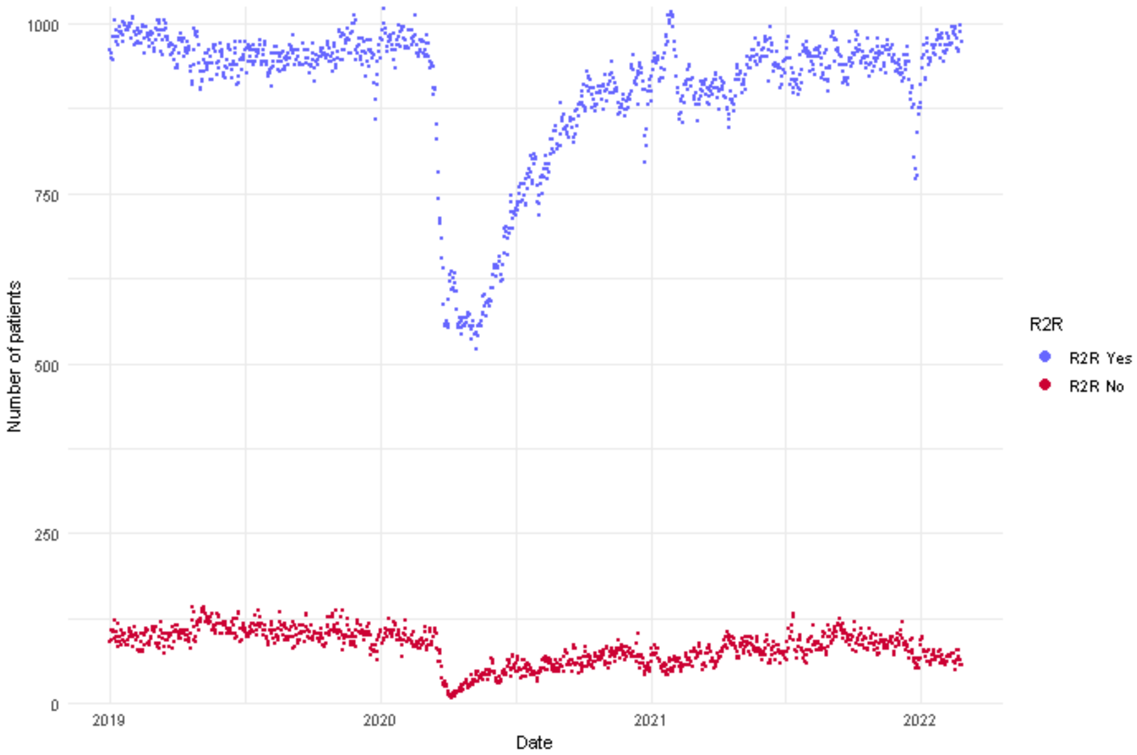

Figure S3. The number of patients meeting or not meeting eR2Rab criteria 01 Jan 2019 - 31 Dec 2021

Legend: Number of patients with (red dot) or without (blue dot) eR2Rab at 00:00 on each day of 2019-2021. The first COVID-19 admission to QEHB occurred on 1st March 2020. The first wave of the pandemic was associated with significant changes resulting in reduced bed occupancy and the majority of admitted patients had a diagnosis of COVID-19
