# Supplementary material for: Variability and performance of NHS England’s ‘reason to reside’ criteria in predicting hospital discharge in acute hospitals in England: a retrospective, observational cohort study
Source: BMJ Open. 2022 Dec 26;12(12):e065862. doi: 10.1136/bmjopen-2022-065862 (PMC9805825; doi:10.1136/bmjopen-2022-065862)
Supplement: Author's manuscript [file bmjopen-2022-065862.draft_revisions.pdf]

BMJ Open is committed to open peer review. As part of this commitment we make the peer review history of every article we publish publicly available.

When an article is published we post the peer reviewers' comments and the authors' responses online. We also post the versions of the paper that were used during peer review. These are the versions that the peer review comments apply to.

The versions of the paper that follow are the versions that were submitted during the peer review process. They are not the versions of record or the final published versions. They should not be cited or distributed as the published version of this manuscript.

BMJ Open is an open access journal and the full, final, typeset and author-corrected version of record of the manuscript is available on our site with no access controls, subscription charges or pay-per-view fees (http://bmjopen.bmj.com).

If you have any questions on BMJ Open's open peer review process please email info.bmjopen@bmj.com

BMJ Open

The variability and performance of NHS England's "Reason to Reside" criteria in predicting hospital discharge in acute hospitals in England. An observational study.

Journal:	*BMJ Open*
Manuscript ID	bmjopen-2022-065862
Article Type:	Original research
Date Submitted by the Author:	23-Aug-2022

Complete List of Authors:	Sapey, Elizabeth; University of Birmingham, PIONEER Data Hub; University Hospitals Birmingham NHS Foundation Trust, Acute Medicine<br>Gallier, Suzy; University Hospitals Birmingham NHS Foundation Trust<br>Evison, Felicity; University Hospitals Birmingham NHS Foundation Trust, Department of Informatics<br>McNulty, David; University Hospitals Birmingham NHS Foundation Trust, Informatics<br>Reeves, Katherine; University Hospitals Birmingham NHS Foundation Trust, Health Informatics<br>Ball, Simon; University Hospitals Birmingham NHS Foundation Trust

Keywords:	Information management < BIOTECHNOLOGY & BIOINFORMATICS, Health policy < HEALTH SERVICES ADMINISTRATION & MANAGEMENT, Quality in health care < HEALTH SERVICES ADMINISTRATION & MANAGEMENT

SCHOLARONE™
Manuscripts

I, the Submitting Author has the right to grant and does grant on behalf of all authors of the Work (as defined in the below author licence), an exclusive licence and/or a non-exclusive licence for contributions from authors who are: i) UK Crown employees; ii) where BMJ has agreed a CC-BY licence shall apply, and/or iii) in accordance with the terms applicable for US Federal Government officers or employees acting as part of their official duties; on a worldwide, perpetual, irrevocable, royalty-free basis to BMJ Publishing Group Ltd ("BMJ") its licensees and where the relevant Journal is co-owned by BMJ to the co-owners of the Journal, to publish the Work in this journal and any other BMJ products and to exploit all rights, as set out in our licence.

The Submitting Author accepts and understands that any supply made under these terms is made by BMJ to the Submitting Author unless you are acting as an employee on behalf of your employer or a postgraduate student of an affiliated institution which is paying any applicable article publishing charge ("APC") for Open Access articles. Where the Submitting Author wishes to make the Work available on an Open Access basis (and intends to pay the relevant APC), the terms of reuse of such Open Access shall be governed by a Creative Commons licence – details of these licences and which Creative Commons licence will apply to this Work are set out in our licence referred to above.

Other than as permitted in any relevant BMJ Author's Self Archiving Policies, I confirm this Work has not been accepted for publication elsewhere, is not being considered for publication elsewhere and does not duplicate material already published. I confirm all authors consent to publication of this Work and authorise the granting of this licence.

For peer review only

The variability and performance of NHS England's "Reason to Reside" criteria in predicting hospital discharge in acute hospitals in England. An observational study.

Elizabeth Sapey[1,3], Suzy Gallier[2,3], Felicity Evison[4], David McNulty[5], Katherine Reeves[6], Simon Ball[7]

1a. PIONEER, HDRUK Health Data Hub in Acute Care, Birmingham, UK. University of Birmingham, Birmingham, UK. ORCiD ID: 0000-0003-3454-5482. e.sapey@bham.ac.uk

1b. Acute Medicine, University Hospitals Birmingham NHS Foundation Trust, Edgbaston, Birmingham, UK.

2. University Hospitals Birmingham NHS Foundation Trust, Edgbaston, Birmingham, UK. Suzy.Gallier@UHB.nhs.uk

3. Joint first authors.

4. University Hospitals Birmingham NHS Foundation Trust, Edgbaston, Birmingham, UK. Felicity.Evison@UHB.nhs.uk

5. University Hospitals Birmingham NHS Foundation Trust, Edgbaston, Birmingham, UK. David.McNulty@UHB.nhs.uk

6. University Hospitals Birmingham NHS Foundation Trust, Edgbaston, Birmingham, UK. Katherine.Reeves@UHB.nhs.uk

7a. Health Data Research UK (HDR-UK) Midland's Physical Site. University of Birmingham, Edgbaston, Birmingham, UK. Institute of Immunology and Immunotherapy, University of Birmingham, Birmingham, UK.

7b Chief Medical Officer, University Hospitals Birmingham NHS Foundation Trust, Edgbaston, Birmingham, UK. Simon.Ball@uhb.nhs.uk

Corresponding author: Elizabeth Sapey. University of Birmingham, Edgbaston, Birmingham, UK, B15 2GW. Institute of Inflammation and Ageing, University of Birmingham, Birmingham, UK. ORCiD ID: 0000-0003-3454-5482. Email: e.sapey@bham.ac.uk Tel: 00 44 121 246 2000

Key words
Health quality, information management, health policy, discharge planning, healthcare decision making

Word count:
Abstract: 290
Main article: 2904

For peer review only

Abstract

Objectives: NHS England (NHSE) advocates "reason to reside" (R2R) criteria to support discharge planning. The proportion of patients without R2R and their rate of discharge are reported daily by acute hospitals in England. R2R has no inter-operable standardised data model (SDM) and its performance has not been validated. We aimed to understand the degree of inter- and intra-centre variation in R2R related metrics reported to NHSE, define a SDM implemented within a single centre Electronic Health Record to generate an eR2R, and evaluate its performance in predicting subsequent discharge.

Design: Retrospective observational cohort study using routinely collected health data.

Setting: 122 NHS Trusts in England for national reporting and a acute hospital in England for local reporting.

Participants: 6,602,706 patient-days were analysed using 3 months national data and 1,039,592 patient-days, using 3 years single centre data.

Main outcome measures: Variability in R2R related metrics reported to NHSE. Performance of eR2R in predicting discharge within 24 hours.

Results: There were high levels of intra and inter-centre variability in R2R related metrics ($p<0.0001$), but not in eR2R. Informedness of eR2R for discharge within 24 hours was low (J-statistic 0.09 – 0.12 across three consecutive years). In those remaining in hospital without eR2R, 61.2% met eR2R criteria on subsequent days (76% within 24 hours), most commonly due to increased NEWS2 (21.9%) or intravenous therapy administration (32.8%).

Conclusions: Reported R2R metrics are highly variable between and within acute Trusts in England. Although case-mix or community care provision may account for some variability, the absence of a SDM prevents standardised reporting. Following the development of a SDM in one acute Trust, the variability reduced. However, the performance of eR2R was poor, prone to change even when negative and unable to meaningfully contribute to discharge planning.

Article Summary

Strengths and weaknesses of the study

Strengths

- The intra and inter-centre variability of R2R reporting was based on national data and included > 6.6M patient bed-days.
- Standardised data model to form eR2R was based on nationally agreed criteria for each clinical question
- All admissions > 24 hours were included for eR2R performance review, reducing bias

Weakness

- eR2R data based on one centre only, albeit one of the largest NHS Trusts nationally serving a diverse population and including >1M patient bed-days.

For peer review only - http://bmjopen.bmj.com/site/about/guidelines.xhtml

Introduction

In 2021 the UK Government published its policy and operating model for hospital discharge and community support within the National Health Service in England (NHSE) [1]. This policy responded to concerns about bed capacity during the COVID-19 pandemic.

A National Audit Office report recognised the potential to release acute hospital beds in 2016, finding that older patients no longer needing acute treatment accounted for 2.7 million NHS hospital bed days per year[2]. The report concluded that a lack of planning delayed discharge, recognising research which highlighted adverse outcomes during prolonged hospital stay[3, 4].

The aforementioned policy mandates using set criteria to identify in-patients in whom discharge home, or to a less acute setting, should be considered. These criteria have been referred to interchangeably, as “reasons to reside” (R2R), “right to remain” or “criteria to reside” (See Table 1a). Since April 2020, NHS hospitals have been required to provide daily reports on the numbers of people leaving hospital, to where, and the reasons for those remaining in hospital. The proportion of in-patients not meeting R2R criteria, and the proportion of patients without R2R discharged that day, are also reported. These metrics are considered to be measures of organisational efficiency.

R2R appears to have emerged heuristically from the clinical experience of those involved in its development. A series of questions are posed that might prompt consideration of individual patients for discharge. However, there are no standardised data definitions, there has been no validation of R2R, no investigation of its role as a clinical decision support tool, or of its value in evaluating hospital performance. A further barrier to evaluating the performance of R2R is that there is no gold standard definition which identifies patients who could be discharged from hospital against which to compare R2R performance. This lack of a reference standard limits, but does not preclude assessment of the validity of a clinical test, provided a ‘fair’ measure of performance can be defined[5]. The set of patients actually discharged in the subsequent 24 hours is one potentially ‘fair’ test of performance of R2R.

For peer review only

Table 1. Reason to Reside (R2R)

1	Requiring ITU or HDU care
2	Requiring oxygen therapy / NIV
3	Requiring intravenous fluids
4	NEWS2 > 3 (clinical judgement required in persons with AF and/or chronic respiratory disease)
5	Diminished level of consciousness where recovery realistic
6	Acute functional impairment in excess of home/community care provision
7	Last hours of life
8	Requiring intravenous medication > bd (including analgesia)
9	Undergone lower limb surgery within 48 hours
10	Undergone throrax-abdominal/pelvic surgery within 72 hours
11	Within 24 hours of an invasive procedure? (with attendant risk of acute life-threatening deterioration)

Legend. The policy and operating model for hospital discharge and community support within the National Health Service in England states that every person on every general ward should be reviewed on a twice daily ward round to determine whether they meet R2R. If the answer to each question is 'no', the policy states that active consideration for discharge to a less acute setting must be made (1).

In the current study, we show the degree of variation in R2R associated metrics reported across centres in England. Secondly, we propose precisely defined, inter-operable, data definitions corresponding to the elements of R2R. This allows for consistent, generalisable analysis. Thirdly, we evaluate the performance of R2R to predict discharge over the subsequent 24 hours.

Methods

This study used unconsented, anonymous health data and all study activity was approved by the East Midlands–Derby REC (reference: 20/EM/0158) and was supported by PIONEER, the Health Data Hub in acute care. All studies activities followed the World Medical Association's Declaration of Helsinki.

National Data

National NHS England data was accessed via The UK Health Facts and Dimensions database[(6)] for all reporting Trusts in England. Assessment of variability in national R2R reporting included data from 29th November 2021 to 20th February 2022. Table S1 of the online supplement provides the names of the Trusts whose data are

For peer review only

presented anonymously. Data were collected daily during the censor period for 121 centres, yielding a total of 10,164 potential data points (centre-days). For each of these, the total number of occupied and unoccupied beds, and the number of patients with no right to reside were extracted. The numbers of patients with right to reside were then calculated by subtracting the number with no right to reside from the total number of occupied beds on that day. The number of General and Acute beds occupied in any given centre, on any given day (in-patients), was used as a surrogate for the number of patients eligible for evaluation using the R2R criteria. Review of the dataset found some missing, and potentially spurious data, which were excluded prior to analysis. This comprised instances where R2R data were not recorded (N=184 data points); where the total numbers of beds were either zero, missing, or clearly spurious (N=37 data points); or where there were more patients with no R2R than the total number of beds (N=3 data points).

Local Data

In-depth analysis of R2R criteria were performed using data from the Queen Elizabeth Hospital Birmingham (QEHB). QEHB is a National Health Service (NHS), urban, adult, acute hospital in England which in 2019 had 1269 beds including 80 level 2/3 intensive care (ICU) beds, an Emergency Department that assesses >300 patients per day, and a mixed secondary and tertiary practice that includes all major adult specialities except for obstetrics and gynaecology. The electronic healthcare record (EHR) at QEHB (PICS, Birmingham Systems) contains time-stamped, structured records that include demography, location, admission and discharge, co-morbidities, physiological measurements supporting NEWS2 and Glasgow Coma Scale, operation noting, prescribing and investigations. The R2R criteria in Table 1 were mapped to computable definitions derived from the EHR (See Table 2), to generate an electronic R2R (eR2R). The OPCS Classification of Interventions and Procedures codes mapped to criterion 9-11 are described in Table S2 of the online supplement. The concept 'acute functional impairment in excess of home/community care provision', had no direct correlate. Safer Nursing Care Tool (SNCT) levels of care were however available[7]. SNCT level 2 and 3 correspond closely with the requirement for HDU or ICU[8]. Level 1a identifies patients requiring enhanced nursing reflecting acuity of illness and Level 1b identifies a group with increased nursing dependency. Level 1b is likely to include those who would and would not be considered to require ongoing care in acute hospital. SNCT level 1 was included in the definition of eR2R in two ways, including (eR2Rab) and excluding (eR2Ra) level 1b, to determine if this affected performance.

For peer review only - http://bmjopen.bmj.com/site/about/guidelines.xhtml

	Flag if…	R2R criterion number
On ITU HDU	listed as being in ITU or HDU ward	1
SNCT Level ≥ 2	Most recent SNCT level in previous 48 hours ≥ 2	1
SNCT Level 1a	Most recent SNCT level in previous 48 hours = 1a	6
SNCT Level 1b	Most recent SNCT level in previous 48 hours = 1b	6
Oxygen therapy/ NIV	oxygen administration or NIV documented in observation chart within previous 24 hours	2
Intravenous fluids	iv fluid administration initiated in previous 24 hours or variable rate insulin infusion administered in previous 24 hours	3
NEWS2	if NEWS2 > 3 within last 24 hrs	4
Diminished consciousness	Glasgow Coma Scale value ≤ 12 in last 24 hours	5
Last hours of life	comfort observation completed current OR End of Life medication bundle administered within last 24 hours	7
Intravenous prescription ≥ tds current (regular not prn)	IV medication prescribed within last 24 hours and frequency ≥ ≥ 3 times per day for regular medication only	8
Intravenous medication administration ≥ tds within 24 hrs	IV medication administered ≥ 3 times within last 24 hours	8
Lower limb surgery within 48hrs	Procedure with relevant OPCS codes in previous 48 hours	9
Thorax-abdominal-pelvic surgery with 72hrs	Procedure with OPCS relevant codes in previous 72 hours	10
Invasive procedure within 24hrs	Procedure with OPCS relevant codes in previous 24 hours	11

Table 2. Data definitions used to operationalise R2R for EHR
Legend. The table describes the data definitions used and the R2R criteria they map to. ITU = intensive care. HDU = High dependency unit. SNCT = Safer Nursing Care Tool. NEWS2 = National Early Warning Score 2. tds = thrice daily. OPCS = OPCS Classification of Interventions and Procedures code which is used to identify the coded clinical entry. All OPCS codes used to identify procedures are listed in Table S2 of the online supplement.

For peer review only

The primary analysis of eR2R was for patients who had been in hospital for more than twenty-four hours at midnight. Discharge over the course of the subsequent twenty-four hours was evaluated. Secondary analyses were undertaken for the set of patients in a bed at 08:00 and at 16.00 to define any change in eR2R performance in these different cross sections of the in-patient population. Three calendar years were analysed separately, to assess the effects of the COVID19 pandemic.

Statistics

Initially, daily numbers of patients with R2R quantified both as absolute numbers and a proportion of the total number of beds, were plotted for national centres and used to calculate between-centre and within-centre variation. These data are analysed as beds occupied at the specified time of day, where the bed inherits the demographics, comorbidities, and other qualities of the occupying patient. This represents the in-patient population in cross-section. This approach was replicated in the local analysis of eR2R: the term patient-day was used to refer to a bed with the qualities of the occupying patient at the time of the analysis. The in-patient

population is described as means of patient-days thereby representing a cross-section of the group. The performance of eR2R as a predictor of remaining in hospital (or absence of eR2R as a predictor of discharge) was reported as a True Positive Rate (TPR) and True Negative Rate (TNR), Positive Predictive Value (PPV), Negative Predictive Value (NPV) and Youden's J statistic (TPR+TNR-1), where positive is remains in hospital and negative is discharge from hospital within 24 hours.

Normally distributed variables are reported as arithmetic means ± standard deviations, with medians and ranges used otherwise. Between-centre variation was assessed by ANOVA. This included a model accounting for day of the week as a fixed effect and the centre as a random effect. All analyses were performed using IBM SPSS 22 (IBM Corp. Armonk, NY), with $p<0.05$ deemed to be indicative of statistical significance throughout

Patient and public involvement

The research question and topic were agreed following patient/public discussion groups about NHSE discharge policies. Patients/public reviewed the data fields included in the study, with the PIONEER Data Trust Committee providing support for the project (a group of patient/public members who review studies using health data[(9)]). A patient/public group have reviewed the results and have written a lay summary for study dissemination to patient groups

<u>Results</u>

R2R reporting in England Nov 20-Feb 21

Across 10,164 available centre-days, accounting for 6,602,706 patient-days, the number of patients reported without R2R as a proportion of in-patients, varied significantly between centres ($p<0.0001$). Individual centre means ranged from 6.7% ± 2.5% to 59.9% ± 13.8% (Figure 1a). There was also marked within-centre variation (Figure 1a), with coefficients of variation (CV) ranging from 8.2% up to 59.3%. Of patients not meeting R2R criteria, the proportion discharged over the following 24 hours, varied significantly between centres ($p<0.0001$). Individual centre means ranged from 14.0% ± 7.4% to 85.8% ± 25.2% (Figure 1b). There was also marked within centre variation, with coefficients of variation ranging from 6.4% up to 83.2%. This variation was not significantly altered by accounting for effects of day of the week (Figure S1 of the online supplement). The proportion of patients without R2R and the proportion of that group discharged within 24 hours, were only weakly correlated (R^2=0.12; Figure S2 of the online supplement).

Performance of eR2R at QEHB

Patients and admissions

For peer review only

Standardised definitions corresponding to the elements of R2R (Table 2) were used to analyse data from QEHB, on 1,214,480 in-patient days, between 01 Jan 2019 – 31 Dec 2021. The demographic and clinical details of that population are summarised in Table 3 which also shows that those meeting the definition of eR2Rab were older and more likely to have one or more co-morbidities than those who did not. Variation in the daily number of patients with or without an eR2R is shown in Figure S3 of the online supplement.

	**All QEHB patient days**	**Meeting eR2Rab**	**Not meeting eR2Rab**
n	1039592	919751 (88.5%)	119841 (11.5%)
**Age in years***: median (IQR)	68 (53-80)	69 (54-81)	63 (48-76)
**Sex* (n, %)**			
Female	488120 (47.0%)	434418 (47.2%)	53702 (44.8%)
Male	546061 (52.5%)	484816 (52.7%)	61245 (51.1%)
Not recorded	5411 (0.5%)	517 (0.1%)	4894 (4.1%)
**Self-reported ethnicity* (n, %)**			
White	784528 (75.5%)	698573 (76.0%)	85955 (71.7%)
Mixed/ Multiple	12983 (1.2%)	11023 (1.2%)	1960 (1.6%)
South Asian/ Asian British	114049 (11.0%)	98903 (10.8%)	15146 (12.6%)
Black/ African/ Caribbean/ Black British	51122 (4.9%)	43991 (4.8%)	7131 (6.0%)
Other ethnic group	19475 (1.9%)	16623 (1.8%)	2852 (2.4%)
Not known	57435 (5.5%)	50638 (5.5%)	6797 (5.7%)
**Co-morbidity count* (n, %)**			
None	196121 (18.9%)	164704 (17.9%)	31417 (26.2%)
1-2	474922 (45.7%)	423200 (46.0%)	51722 (43.2%)
3 or more	368549 (35.5%)	331847 (36.1%)	36702 (30.6%)
**Morbidities (n, %)**			
Hypertension*	492160 (47.3%)	439930 (47.8%)	52230 (43.6%)
Cerebrovascular disease*	159316 (15.3%)	147676 (16.1%)	11640 (9.7%)
Atrial fibrillation*	224501 (21.6%)	204458 (22.2%)	20043 (16.7%)
Ischaemic heart disease, angina, myocardial infarct*	198480 (19.1%)	173708 (18.9%)	24772 (20.7%)
Diabetes (type 1 and 2)*	271505 (26.1%)	242328 (26.3%)	29177 (24.3%)
Asthma*	103679 (10.0%)	91136 (9.9%)	12543 (10.5%)
COPD*	112731 (10.8%)	103882 (11.3%)	8849 (7.4%)
Interstitial Lung Disease*	2533 (0.2%)	2380 (0.3%)	153 (0.1%)
Chronic Kidney Disease*	198052 (19.1%)	178284 (19.4%)	19768 (16.5%)
Any active Malignancy *	215959 (20.8%)	194419 (21.1%)	21540 (18.0%)
Dementia (all types)*	65272 (6.3%)	61324 (6.7%)	3948 (3.3%)
**English Indices of deprivation**			
1	430114 (41.4%)	382132 (41.5%)	47982 (40.0%)
2	222478 (21.4%)	197999 (21.5%)	24479 (20.4%)
3	178565 (17.2%)	158047 (17.2%)	20518 (17.1%)
4	107747 (10.4%)	96115 (10.5%)	11632 (9.7%)
5	75854 (7.3%)	67296 (7.3%)	8558 (7.1%)
Not recorded	24834 (2.4%)	18162 (2.0%)	6672 (5.6%)
**Care escalation to ITU (n, %)**	101017 (9.7%)	93080 (10.1%)	7937 (6.6%)

For peer review only

Table 3. Demographics of patients meeting and not meeting R2R criteria on presentation to QEHB in the censor period.
Legend. Data is number (percentage) of patients in a bed at 00:00. Ethnicity was self-reported. Medical conditions were physician confirmed and checked against admission and linked primary care notes. English Indices of deprivation were calculated using postcode. *Significant difference between meeting and not meeting eR2Rab (p<0.05 in univariate analysis)

Criteria contributing to eR2R

Given the potential for the COVID19 pandemic to affect R2R, calendar years were analysed separately. The number of patients meeting any given eR2R criterion are shown in Table 4a. The progressive contribution of different elements of the definition of eR2R assessed daily in a modified Consort table, are summarised in Table 4b. The proportion of patients not meeting eR2R criteria exhibited relatively little day to day variation in 2019 (eR2Rab, CV = 11.2%; eR2Ra, CV = 6.3%), although somewhat higher in the context of case mix variation consequent upon peaks of patients admitted with COVID-19 in 2020 (eR2Rab, CV = 23.3%; eR2Ra, CV = 14.4%) and 2021 (eR2Rab, CV=17.1%; eR2Ra, CV = 9.9%). The criteria contributing most to eR2R status included acuity level (NEWS2 >3), SNCT level nursing requirement, being on intensive care and requiring intravenous medications or fluids.

**Year**	**2019**	**2020**	**2021**
**Criterion**	n (%)	n (%)	n (%)
ICU	22899 (6.1)	20326 (6.7)	21305
TAP surgery 72Hrs	3783 (1.0)	3010 (1.0)	3974
Lower limb surgery 48Hrs	285 (0.1)	252 (0.1)	221 (0.1)
Invasive surgery 24Hrs	1861 (0.5)	1613 (0.5)	1988 (0.6)
NEWS2 > 3 24hrs	93501 (24.8)	85123 (27.9)	97722 (27.3)
O2 Treatment 24Hrs	77949 (20.7)	69355 (22.7)	77202 (21.6)
Insulin Infusion 24Hrs	10951 (2.9)	10860 (3.6)	12496 (3.5)
IV Fluids 24Hrs	79802 (21.2)	71376 (23.4)	80246 (22.4)
IV medication administered in last 24hrs >= tds	95034 (25.2)	81174 (26.6)	91573 (25.6)
IV medication prescribed in last 24Hrs >= tds	21543 (5.7)	17866 (5.9)	19249 (5.4)
SNCT Dependency 1a, 2, 3	99139 (26.3)	72226 (23.7)	88832 (54.8)
COMA Score <=12 in last 24Hrs	6594 (1.8)	6448 (2.1)	6664 (1.9)
End of Life care definition met in last 24Hrs	5359 (1.4)	4747 (1.6)	5075 (1.4)
SNCT Dependency 1b	172659 (45.8)	160380 (52.5)	179527 (50.2)
Total number of patient days	376684	305254	357654

Table 4a. The number (percentage) of patient-days on which each eR2R data definition was met
Legend. The number (percentage) of patient days on which each eR2R definition was met. The population was in-patients at 24.00 with length of stay ≥ 24 hours.

For peer review only

Year	2019	2020	2021
**Criterion**	Mean % (SD)	Mean % (SD)	Mean % (SD)
ICU	6.1% (0.44)	7.1% (3.10)	6.0% (2.16)
TAP surgery 72Hrs	0.7% (0.35)	0.7% (0.37)	0.8% (0.45)
Lower limb surgery 48Hrs	0.1% (0.07)	0.1% (0.11)	0.1% (0.08)
Invasive surgery 24Hrs	0.2% (0.15)	0.2% (0.18)	0.2% (0.15)
NEWS2 > 3 24hrs	24.2% (2.28)	27.5% (3.82)	26.6% (3.64)
O2 Treatment 24Hrs	4.0% (0.61)	3.9% (0.72)	3.6% (0.68)
Insulin Infusion 24Hrs	0.5% (0.24)	0.6% (0.28)	0.5% (0.23)
IV Fluids 24Hrs	8.8% (1.09)	9.5% (1.37)	9.6% (1.24)
IV Medication Admin 24Hrs >= tds	7.7% (1.05)	7.4% (1.29)	7.5% (1.17)
IV Medication Prescribed 24Hrs	0.7% (0.28)	0.6% (0.29)	0.6% (0.27)
SNCT Dependency 1a, 2, 3	8.8% (1.42)	6.7% (1.21)	7.8% (1.12)
COMA Score <=12 24Hrs	0.0% (0.05)	0.0% (0.08)	0.0% (0.06)
End of Life 24Hrs	0.5% (0.24)	0.4% (0.27)	0.4% (0.19)
SNCT Dependency 1b	24.5% (1.88)	25.5% (3.53)	25.3% (2.59)
No eR2Rab total	13.3% (1.50)	9.8% (2.29)	10.9% (1.87)
No eR2Ra total	37.8% (2.38)	35.3% (5.08)	36.2% (3.60)

Table 4b. A phased analysis undertaken for each day and presented as a modified Consort Diagram.

Table 4b: A phased analysis undertaken for each day and presented as a modified Consort Diagram. The progressive contribution of each element to the definition of eR2R was calculated as proportion of the whole population. These were aggregated by calendar year. The order of the phased analysis was determined by the researchers to be that which was most informative, and which placed objective definitions earlier. SNCT dependency is a global nursing assessment and therefore was placed last.

Informedness of eR2R for discharge in the next 24 hours

For the outcome discharge (remain -) / no discharge (remain +) within 24 hours, across the 3 different years, the eR2Ra TPR lay between 0.63 and 0.65, TNR between 0.46 and 0.47, the PPV was 0.91 and NPV between 0.12 and 0.15; the eR2Rab TPR lay between 0.88 and 0.91, TNR between 0.18 and 0.24, the PPV between 0.90 and 0.91 and NPV between 0.18 and 0.20 (Table 5). The J statistic for both definitions lay between 0.09-0.12. In secondary analyses based upon the in-patient population at 08.00 and at 16.00 the J-statistic ranged between 0.10-0.14 and 0.10-0.15 respectively (Tables S3a and S3b of the online supplement).

For peer review only

A.

	2019	Remain		
		Yes (+)	No (-)	Total
eR2Ra	Yes (+)	213,382	20,845	234,227
	No (-)	124,874	17,583	142,457
	Total	338,256	38,428	376,684
	**2020**	**Remain**		
		Yes (+)	No (-)	Total
eR2Ra	Yes (+)	177,065	18,292	195,357
	No (-)	93,947	15,950	109,897
	Total	271,012	34,242	305,254
	**2021**	**Remain**		
		Yes (+)	No (-)	Total
eR2Ra	Yes (+)	208,068	20,084	228,152
	No (-)	112,007	17,495	129,502
	Total	320,075	37,579	357,654

B.

	2019	Remain		
		Yes (+)	No (-)	Total
eR2Rab	Yes (+)	297,172	29,372	326,544
	No (-)	41,084	9,056	50,140
	Total	338,256	38,428	376,684
	**2020**	**Remain**		
		Yes (+)	No (-)	Total
eR2Rab	Yes (+)	246,461	28,026	274,487
	No (-)	24,551	6,216	30,767
	Total	271,012	34,242	305,254
	**2021**	**Remain**		
		Yes (+)	No (-)	Total
eR2Rab	Yes (+)	288,384	30,336	318,720
	No (-)	31,691	7,243	38,934
	Total	320,075	37,579	357,654

Table 5. Contingency tables showing the number of patients meeting criteria for (A) eR2Ra and (B) eR2ab

Legend. The tables show numbers of patients meeting R2R criteria and the corresponding number of patients who remain in hospital over the next 24 hours or do not (were discharged), for the in-patient population at 00:00. For eR2Ra, the TPR varied between 0.62-0.65 and TNR 0.46-0.51, across 3 different years and 3 different time points. For eR2Ra, the TPR varied between 0.87-0.91 and TNR 0.18-0.25, across 3 different years and 3 different time points. Table S3 of the online supplement showsthe same data for the in-patient population at 16:00.

For peer review only

In-patients not meeting eR2R

The demographic and clinical details of patient who did not meet the eR2Rab definition, stratified by discharge in the subsequent 24 hours are shown in Table S4 of the online supplement. For patient-days on which discharge occurred within 24 hours, there was significantly higher representation of those with no documented co-morbidities 29.2% vs 24.0% ($p<0.0001$). In those that remained in hospital, 61.2% met eR2R criteria on subsequent days (76% within the next 24 hours). Of all those that remained, 21.9% acquired a NEWS2 > 3, 32.8% received iv fluids or drugs > 3 times / day and 1.9% were admitted to ICU.

Discussion

Assessment of an individual patient's R2R has been promoted as a tool to improve the identification of those who could be discharged from acute hospitals in England. The proportion of in-patients with R2R and their rate of discharge has then been used to evaluate the operational efficiency of acute hospitals and their adjacent

health and social care system[1, 10]. This paper presents findings to suggest that as currently constituted, R2R is of limited value for these purposes.

The high levels of variation in R2R related metrics, within and between centres in England, has been attributed to variation in case mix and operational efficiency[11]. However, such extremes of variation are not observed in other metrics that use established data standards. Furthermore, the proportion of patients not meeting R2R criteria correlates poorly with their rate of discharge over the subsequent 24 hours, whereas one might anticipate that such closely related measures of operational efficiency would reflect one another. These findings are most obviously accounted for by the fact that R2R does not constitute a semantic data model. It is therefore susceptible to differing interpretation by individuals and centres. This applies to all the concepts described by R2R, but most obviously those that are necessarily subjective, such as 'acute functional impairment in excess of home/community care provision' and 'diminished level of consciousness where recovery is realistic'[12, 13].

We therefore developed machine readable data definitions corresponding to each concept, allowing consistent analysis of R2R at scale, using data derived from the EHR in our centre. The SNCT is a global nursing assessment of acuity and dependency that was developed to guide workforce deployment. It is regularly recorded within the EHR at our centre. Because Level 1b describes a group of patients who are highly dependent upon nursing care for daily activities, this was mapped onto the R2R concept 'acute functional impairment in excess of home/community care provision'. However, since the definition of level 1b could include a group of patients suitable for discharge to a less acute setting, two definitions or eR2R were tested, with and without SNCT 1b. Our analysis is therefore likely to represent two extremes of inclusion of patients with acute functional impairment.

Within centre variation in eR2R was low, consistent with it minimising individual interpretation of each data element. eR2R was a poor predictor of discharge within 24 hours[14]. Youden's Index was consistently <0.15 across 3 calendar years, 3 different times of day and two eR2R definitions. For a dichotomous test such as eR2R, a Youden's Index >0.50 is generally considered the empirical benchmark for a test to support clinical decision making[15]. eR2R is therefore unsuited to the provision of clinical decision support tool for discharge. It does not define a sub-population on which to assess discharge performance[16]. The limitations of R2R are not entirely surprising, given the need to interpret concepts that are not semantically defined. Although addressed by eR2R, it nevertheless remains a simple series of binary responses to questions that have not been validated for the purpose of discharge prediction. For example, NEWS2 was validated as an acuity score to quantify physiological instability on initial presentation to hospital[17]. It was not developed and has not been validated, as a triage tool to assess fitness to leave hospital, at any threshold.

Importantly, more than half of those who remain in hospital without eR2R, subsequently acquired eR2R. This group of patients were older and had multiple long-term health conditions, suggesting that there were clinical grounds for that decision, albeit undefined. This sub-population requires further study.

For peer review only

There are limitations to our analysis. The eR2R was assessed in only one centre, albeit one that serves a diverse, multi-ethnic, urban population, in which more than 1.2 million patient days were assessed. Patients admitted for < 24 hours at the time of analysis were excluded, to allow clinical decisions to be made and executed. The first day post-admission is a highly dynamic situation, with frequent clinical review; a setting in which this embodiment of clinical decision support is arguably less relevant. Another, more intrinsic problem, is that there is no gold standard by which to define all patients suitable for discharge, so that actual discharge was used as a fair test when evaluating the performance of eR2R(18). This assumes that patients actually discharged are part of a continuous population of all those who could be discharged. It is also the case that each eR2R data element could be defined in different ways, however each definition would relate to that used, so that the performance of one model would be informed by the other. For example, the 24-hour retrospective time horizon for most evaluations could be altered, but the later model would relate directly to the former.

It is important to validate and evaluate tests within their intended setting. The effects of embedding new care pathways or tools within clinical service delivery, without appropriate evaluation, are increasingly described. There is significant opportunity for unintended consequences to arise from the implementation of poorly considered clinical decision support(19), particularly when there is competition for clinical resource. This has been recently discussed for NEWS2(20), sepsis alerting and COVID-19 virtual wards(21). R2R has been endorsed and adopted but without validation or consideration of the unintended consequences of its application. This is not to contend that a significant number of in-patients could not be discharged earlier, simply that there is no evidence that R2R can support clinical decision making. The collective limitations of R2R identified are likely to account for variation in nationally reported metrics which are difficult to explain.

Our study highlights the need for reproducible standardised data definitions to support both implementation and validation of any tool that purports to support clinical decision making. Further research should focus on building, validating and refining tools to inform clinical decisions.

For peer review only

Figure Legends

Figure 1. National reporting of R2R criteria

Legend. The proportion of patients with no R2R (Figure 1A) and of that group the proportion of patients discharged within 24 hours (Figure 1B) reported to SDCS from 29 Nov 2021 – 20 Feb 2022 across 121 centres. Each dot represents result for a single centre-day.

Acknowledgements and funding.

This work was funded by HDR-UK (PIONEER2019) and was supported by the PIONEER Data Hub (see www.pioneerdatahub.co.uk). It was supported by the PIONEER patient and public advisory group and Data Trust Committee.

Transparency statement

Professor Ball (the manuscript's guarantor) affirms that the manuscript is an honest, accurate, and transparent account of the study being reported; that no important aspects of the study have been omitted; and that any discrepancies from the study as originally planned (and, if relevant, registered) have been explained.

Data Sharing Statement

The anonymised dataset used for analysis is available upon reasonable request from the PIONEER Data Hub on submission of a data request form, see www.pioneerdatahub.co.uk for a copy of the form and processes for data access.

Conflicts of interest

Felicity Evison, David McNulty, Katherine Reeves have no relevant conflicts of interest. Suzy Gallier reports grant funding from HDR-UK. Elizabeth Sapey reports grant funding from HDR UK, Innovate UK, MRC, NIHR, British Lung Foundation and Alpha 1 Foundation. Simon Ball reports funding from HDR-UK.

Author Contributions

Simon Ball and Elizabeth Sapey conceived the study, Suzy Gallier, Felicity Evison, David McNulty, Katherine Reeves conducted data analysis. Elizabeth Sapey and Suzy Gallier wrote the first draft of the study. All authors contributed to the study manuscript. Simon Ball is senior author and manuscript guarantor.

Ethics Approval

This study used unconsented, anonymous health data and all study activity was approved by the East Midlands–Derby REC (reference: 20/EM/0158) and was supported by PIONEER, the Health Data Hub in acute care.   All studies activities followed the World Medical Association's Declaration of Helsinki.

For peer review only

References

1. UK Government. Hospital Discharge and Community Support: Policy and Operating Model. https://assetspublishingservicegovuk/government/uploads/system/uploads/attachment_data/file/1026672/hospital-discharge-and-community-support-policy-and-operating-model-oct-2021pdf. 2021;Date of access 13th Feb 2022.
2. National Audit Office. Discharging older patients from hospital. Report by the Comptroller and Auditor General. https://wwwnaoorguk/wp-content/uploads/2015/12/Discharging-older-patients-from-hospitalpdf. 2016;Date of access: 12th February 2022.
3. Hartley P, Costello P, Fenner R, Gibbins N, Quinn É, Kuhn I, et al. Change in skeletal muscle associated with unplanned hospital admissions in adult patients: A systematic review and meta-analysis. PloS one. 2019;14(1):e0210186-e.

4. Mudge AM, McRae P, Hubbard RE, Peel NM, Lim WK, Barnett AG, et al. Hospital-Associated Complications of Older People: A Proposed Multicomponent Outcome for Acute Care. J Am Geriatr Soc. 2019;67(2):352-6.
5. Rutjs A, Reitsma J, Coomarasamy A, Khan K, Bossuyt P. Evaluation of diagnostic tests when there is no gold standard.
A review of methods. Health Technology Assessment. 2007;11:1 - 72.
6. UK Health Facts. UK Health Facts Database. https://factsanddimensionscouk/ukhf. 2022;Date of access 22nd February 2022.
7. NHS England. Safer nursing care tool. https://wwwenglandnhsuk/nursingmidwifery/safer-staffing-nursing-and-midwifery/safer-nursing-care-tool/. 2021;Date of access: 1st February 2022.
8. National Health Service. CRITICAL CARE LEVEL, The level of critical care provided during a Hospital Provider Spell. https://wwwdatadictionarynhsuk/attributes/critical_care_levelhtml. 2021;Date of access; 22nd February 2022.
9. Gallier S, Price G, Pandya H, McCarmack G, James C, Ruane B, et al. Infrastructure and operating processes of PIONEER, the HDR-UK Data Hub in Acute Care and the workings of the Data Trust Committee: a protocol paper. BMJ Health Care Inform. 2021;28(1).
10. Department of Health and Social Care. QUICK GUIDE: DISCHARGE TO ASSESS. https://wwwnhsuk/NHSEngland/keogh-review/Documents/quick-guides/Quick-Guide-discharge-to-accesspdf. 2016;Date of access: 10th February 2022.
11. Vaughan L, Bardlsey M, Bell D, Davies M, Goddard A, Imison C, et al.
Models of generalist and specialist care in smaller hospitals in England: a mixed-methods study. NIHR Journals Library; 2021 Feb (Health Services and Delivery Research, No 94) Available from: https://wwwncbinlmnihgov/books/NBK568036/ doi: 103310/hsdr09040. 2021;Date of access 24th March 2022.
12. de Lange E, Verhaak P.F., van der Meer K. Prevalence, presentation and prognosis of delirium in older people in the population, at home and in long term care: a review. Geriatric Psychiatry. 2012;28(1099-1166 (Electronic)):127 - 34.
13. Morandi A, Davis D, Bellelli G, Arora RC, Caplan GA, Kamholz B, et al. The Diagnosis of Delirium Superimposed on Dementia: An Emerging Challenge. J Am Med Dir Assoc. 2017;18(1):12-8.
14. Youden WJ. Index for rating diagnostic tests. Cancer. 1950;3(0008-543X (Print)):32 - 5.
15. Hilden J, Glasziou P. Regret graphs, diagnostic uncertainty and Youden's Index. Stat Med. 1996;15(0277-6715 (Print)):969-86.
16. Schisterman EF, Faraggi D, Reiser B, Hu J. Youden Index and the optimal threshold for markers with mass at zero. Stat Med. 2008;27(2):297-315.
17. Royal College of Physicians. National early warning score (news) 2: standardising the assessment of acute-illness severity in the NHS. updated report of a working Party. https://wwwrcplondonacuk/projects/outputs/national-early-warning-score-news-2. 2017;Date of access: 14th February 2022.
18. Umemneku Chikere CM, Wilson K, Graziadio S, Vale L, Allen AJ. Diagnostic test evaluation methodology: A systematic review of methods employed to evaluate diagnostic tests in the absence of gold standard – An update. PLOS ONE. 2019;14(10):e0223832.
19. Liu X, Glocker B, McCradden MM, Ghassemi M, Denniston AK, Oakden-Rayner L. The medical algorithmic audit. The Lancet Digital Health.
20. Pankhurst T, Sapey E, Gyves H, Evison F, Gallier S, Gkoutos G, et al. Evaluation of NEWS2 response thresholds in a retrospective observational study from a UK acute hospital. BMJ Open. 2022;12(2):e054027.

For peer review only

21. Gallier S, Atkin C, Reddy-Kolanu V, Parekh D, Zou X, Evison F, et al. Applying a COVID Virtual Ward model, assessing patient outcomes and staff workload. Acute Med. 2021;20(4):266-75.

For peer review only

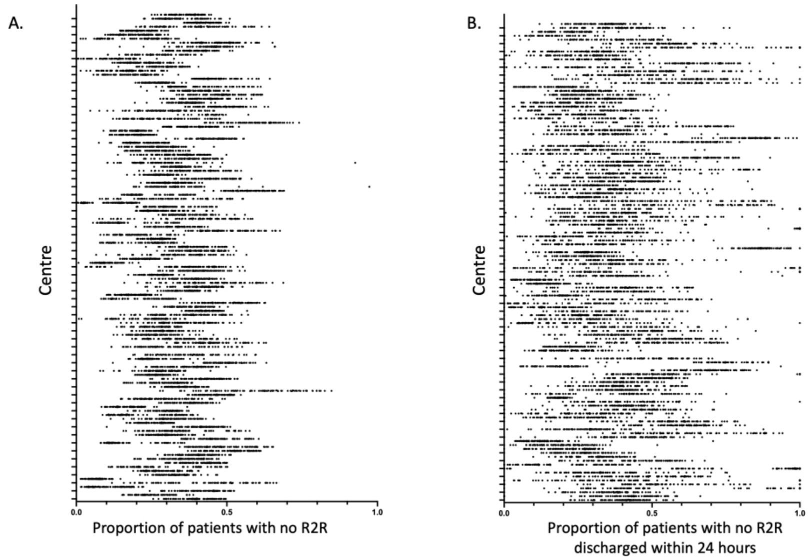

Figure 1. National reporting of R2R criteria

Legend. The proportion of patients with no R2R (Figure 1A) and of that group the proportion of patients discharged within 24 hours (Figure 1B) reported to SDCS from 29 Nov 2021 – 20 Feb 2022 across 121 centres. Each dot represents result for a single centre-day.

338x190mm (225 x 225 DPI)

Online Supplement: The variability and performance of NHS Englands' "Reason to Reside" criteria in predicting hospital discharge in acute hospitals in England

Online Tables

National Trusts	
•Airedale NHS Foundation Trust<br>•Ashford And St Peter's Hospitals NHS Foundation Trust<br>•Barking, Havering And Redbridge University Hospitals NHS Trust<br>•Barnsley Hospital NHS Foundation Trust<br>•Barts Health NHS Trust<br>•Bedfordshire Hospitals NHS Foundation Trust<br>•Blackpool Teaching Hospitals NHS Foundation Trust<br>•Bolton NHS Foundation Trust<br>•Bradford Teaching Hospitals NHS Foundation Trust<br>•Buckinghamshire Healthcare NHS Trust<br>•Calderdale And Huddersfield NHS Foundation Trust<br>•Cambridge University Hospitals NHS Foundation Trust<br>•Chelsea And Westminster Hospital NHS Foundation Trust<br>•Chesterfield Royal Hospital NHS Foundation Trust<br>•Countess Of Chester Hospital NHS Foundation Trust<br>•County Durham And Darlington NHS Foundation Trust<br>•Croydon Health Services NHS Trust<br>•Dartford And Gravesham NHS Trust<br>•Doncaster And Bassetlaw Teaching Hospitals NHS Foundation Trust<br>•Dorset County Hospital NHS Foundation Trust<br>•East And North Hertfordshire NHS Trust<br>•East Cheshire NHS Trust<br>•East Kent Hospitals University NHS Foundation Trust<br>•East Lancashire Hospitals NHS Trust<br>•East Suffolk And North Essex NHS Foundation Trust<br>•East Sussex Healthcare NHS Trust<br>•Epsom And St Helier University Hospitals NHS Trust<br>•Frimley Health NHS Foundation Trust<br>•Gateshead Health NHS Foundation Trust<br>•George Eliot Hospital NHS Trust<br>•Gloucestershire Hospitals NHS Foundation Trust<br>•Great Western Hospitals NHS Foundation Trust<br>•Guy's And St Thomas' NHS Foundation Trust<br>•Hampshire Hospitals NHS Foundation Trust<br>•Harrogate And District NHS Foundation Trust<br>•Homerton University Hospital NHS Foundation Trust<br>•Hull University Teaching Hospitals NHS Trust<br>•Imperial College Healthcare NHS Trust<br>•Isle Of Wight NHS Trust<br>•James Paget University Hospitals NHS Foundation Trust<br>•Kettering General Hospital NHS Foundation Trust<br>•King's College Hospital NHS Foundation Trust<br>•Kingston Hospital NHS Foundation Trust<br>•Lancashire Teaching Hospitals NHS Foundation Trust	•Salisbury NHS Foundation Trust<br>•Sandwell And West Birmingham Hospitals NHS Trust<br>•Sheffield Teaching Hospitals NHS Foundation Trust<br>•Sherwood Forest Hospitals NHS Foundation Trust<br>•Somerset NHS Foundation Trust<br>•South Tees Hospitals NHS Foundation Trust<br>•South Tyneside And Sunderland NHS Foundation Trust<br>•South Warwickshire NHS Foundation Trust<br>•Southport And Ormskirk Hospital NHS Trust<br>•St George's University Hospitals NHS Foundation Trust<br>•St Helens And Knowsley Teaching Hospitals NHS Trust<br>•Stockport NHS Foundation Trust<br>•Surrey And Sussex Healthcare NHS Trust<br>•Tameside And Glossop Integrated Care NHS Foundation Trust<br>•The Dudley Group NHS Foundation Trust<br>•The Hillingdon Hospitals NHS Foundation Trust<br>•The Newcastle Upon Tyne Hospitals NHS Foundation Trust<br>•South Warwickshire NHS Foundation Trust<br>•Southport And Ormskirk Hospital NHS Trust<br>•St George's University Hospitals NHS Foundation Trust<br>•St Helens And Knowsley Teaching Hospitals NHS Trust<br>•Stockport NHS Foundation Trust<br>•Surrey And Sussex Healthcare NHS Trust<br>•Tameside And Glossop Integrated Care NHS Foundation Trust<br>•The Dudley Group NHS Foundation Trust<br>•The Hillingdon Hospitals NHS Foundation Trust<br>•The Newcastle Upon Tyne Hospitals NHS Foundation Trust<br>•The Princess Alexandra Hospital NHS Trust<br>•The Queen Elizabeth Hospital, King's Lynn, NHS Foundation Trust<br>•The Rotherham NHS Foundation Trust<br>•The Royal Wolverhampton NHS Trust<br>•The Shrewsbury And Telford Hospital NHS Trust<br>•Torbay And South Devon NHS Foundation Trust<br>•United Lincolnshire Hospitals NHS Trust<br>•University College London Hospitals NHS Foundation Trust

For peer review only

•Leeds Teaching Hospitals NHS Trust<br>•Lewisham And Greenwich NHS Trust<br>•Liverpool University Hospitals NHS Foundation Trust<br>•London North West University Healthcare NHS Trust<br>•Maidstone And Tunbridge Wells NHS Trust<br>•Manchester University NHS Foundation Trust<br>•Medway NHS Foundation Trust<br>•Mid And South Essex NHS Foundation Trust<br>•Mid Cheshire Hospitals NHS Foundation Trust<br>•Mid Yorkshire Hospitals NHS Trust<br>•Milton Keynes University Hospital NHS Foundation Trust<br>•Norfolk And Norwich University Hospitals NHS Foundation Trust<br>•North Bristol NHS Trust<br>•North Cumbria Integrated Care NHS Foundation Trust<br>•North Middlesex University Hospital NHS Trust<br>•North Tees And Hartlepool NHS Foundation Trust<br>•North West Anglia NHS Foundation Trust<br>•Northampton General Hospital NHS Trust<br>•Northern Care Alliance NHS Foundation Trust<br>•Northern Devon Healthcare NHS Trust<br>•Northern Lincolnshire And Goole NHS Foundation Trust<br>•Northumbria Healthcare NHS Foundation Trust<br>•Nottingham University Hospitals NHS Trust<br>•Oxford University Hospitals NHS Foundation Trust<br>•Portsmouth Hospitals University National Health Service Trust<br>•Royal Berkshire NHS Foundation Trust<br>•Royal Cornwall Hospitals NHS Trust<br>•Royal Devon And Exeter NHS Foundation Trust<br>•Royal Free London NHS Foundation Trust<br>•Royal Surrey County Hospital NHS Foundation Trust<br>•Royal United Hospitals Bath NHS Foundation Trust	•University Hospital Southampton NHS Foundation Trust<br>•University Hospitals Birmingham NHS Foundation Trust<br>•University Hospitals Bristol And Weston NHS Foundation Trust<br>•University Hospitals Coventry And Warwickshire NHS Trust<br>•University Hospitals Dorset NHS Foundation Trust<br>•University Hospitals Of Derby And Burton NHS Foundation Trust<br>•University Hospitals Of Leicester NHS Trust<br>•University Hospitals Of Morecambe Bay NHS Foundation Trust<br>•University Hospitals Of North Midlands NHS Trust<br>•University Hospitals Plymouth NHS Trust<br>•University Hospitals Sussex NHS Foundation Trust<br>•Walsall Healthcare NHS Trust<br>•Warrington And Halton Teaching Hospitals NHS Foundation Trust<br>•West Hertfordshire Hospitals NHS Trust<br>•West Suffolk NHS Foundation Trust<br>•Whittington Health NHS Trust<br>•Wirral University Teaching Hospital NHS Foundation Trust<br>•Worcestershire Acute Hospitals NHS Trust<br>•Wrightington, Wigan And Leigh NHS Foundation Trust<br>•Wye Valley NHS Trust<br>•Yeovil District Hospital NHS Foundation Trust<br>•York And Scarborough Teaching Hospitals NHS Foundation Trust

Table S1. The names of the Hospital Trusts included in the national R2R reporting analysis
Legend. Data is presented anonymously

For peer review only

Procedure	OPCS Codes
Lower limb surgery within 48hrs	See Online supplement xls.
Thorax-abdominal-pelvic surgery with 72hrs	See Online supplement xls.
Invasive procedure within 24hrs	See Online supplement xls.

Table S2. Codes used to identify surgical interventions.
Legend. OPCS = OPCS Classification of Interventions and Procedures code used to identify the coded clinical entry.

	**2019**	**Remain**		
		Yes (+)	No (-)	Total
**eR2Ra**	Yes (+)	214,613	21,333	235,946
	No (-)	128,470	19,270	147,740
	Total	343,083	40,603	383,686
	**2020**	**Remain**		
		Yes (+)	No (-)	Total
**eR2Ra**	Yes (+)	177,852	18,283	196,135
	No (-)	97,119	17,606	114,725
	Total	274,971	35,889	310,860
	**2021**	**Remain**		
		Yes (+)	No (-)	Total
**eR2Ra**	Yes (+)	208,449	19,989	228,438
	No (-)	115,111	19,075	134,186
	Total	323,560	39,064	362,624

	**2019**	**Remain**		
		Yes (+)	No (-)	Total
**eR2Rab**	Yes (+)	301,018	30,176	331,194
	No (-)	42,065	10,427	52,492
	Total	343,083	40,603	383,686
	**2020**	**Remain**		
		Yes (+)	No (-)	Total
**eR2Rab**	Yes (+)	249,964	28,636	278,600
	No (-)	25,007	7,253	32,260
	Total	274,971	35,889	310,860
	**2021**	**Remain**		
		Yes (+)	No (-)	Total
**eR2Rab**	Yes (+)	291,576	30,847	322,423
	No (-)	31,984	8,217	40,201
	Total	323,560	39,064	362,624

Table S3a. Contingency tables showing the number of patients meeting criteria for eR2Ra and eR2ab and the corresponding number of patients who remain in hospital over the next 24 hours or do not (were discharged), for the in-patient population at 08.00.

For peer review only

	2019	Remain		
		Yes (+)	No (-)	Total
eR2Ra	Yes (+)	214,005	19,919	233,924
	No (-)	129,543	18,465	148,008
	Total	343,548	38,384	381,932
	**2020**	**Remain**		
		Yes (+)	No (-)	Total
eR2Ra	Yes (+)	178,709	17,343	196,052
	No (-)	98,123	17,692	115,815
	Total	276,832	35,035	311,867
	**2021**	**Remain**		
		Yes (+)	No (-)	Total
eR2Ra	Yes (+)	211,080	19,105	230,185
	No (-)	116,893	19,616	136,509
	Total	327,973	38,721	366,694

	2019	Remain		
		Yes (+)	No (-)	Total
eR2Rab	Yes (+)	299,551	28,334	327,885
	No (-)	43,997	10,050	54,047
	Total	343,548	38,384	381,932
	**2020**	**Remain**		
		Yes (+)	No (-)	Total
eR2Rab	Yes (+)	250,507	27,672	278,179
	No (-)	26,325	7,363	33,688
	Total	276,832	35,035	311,867
	**2021**	**Remain**		
		Yes (+)	No (-)	Total
eR2Rab	Yes (+)	294,260	30,038	324,298
	No (-)	33,713	8,683	42,396
	Total	327,973	38,721	366,694

Table S3b. Contingency tables showing the number of patients meeting criteria for eR2Ra and eR2ab and the corresponding number of patients who remain in hospital over the next 24 hours or do not (were discharged), for the in-patient population at 16.00.

For peer review only

Population at 00:00	Not meeting eR2Rab criteria and discharged in subsequent 24 hours	Not meeting eR2Rab criteria and not discharged
n	22515	97326
**Age in years**: median (IQR)	60(45-74)	64(49-77)
**Sex (n, %)**	.	
Female	10833 (48.1%)	45345 (46.6%)
Male	11682 (51.9%)	51981 (53.4%)
**Self-reported ethnicity (n, %)**		
White	15761 (70.0%)	70194 (72.1%)
Mixed/ Multiple	411 (1.8%)	1549 (1.6%)
Asian/ Asian British	2952 (13.1%)	12194 (12.5%)
Black/ African/ Caribbean/ Black British	1274 (5.7%)	5857 (6.0%)
Other ethnic group	567 (2.5%)	2285 (2.3%)
Not known	1550 (6.9%)	5247 (5.4%)
**Co-morbidity count (n, %)**		
None	6544 (29.1%)	24873 (25.6%)
1-2	10321 (45.8%)	41401 (42.5%)
3 or more	5650 (25.1%)	31052 (31.9%)
**Morbidities (n, %)**		
Hypertension	9168 (40.7%)	43062 (44.2%)
Cerebrovascular disease	1512 (6.7%)	10128 (10.4%)
Atrial fibrillation	2947 (13.1%)	17096 (17.6%)
Ischaemic heart disease, angina, myocardial infarct	3810 (16.9%)	20962 (21.5%)
Diabetes (type 1 and 2)	4809 (21.4%)	24368 (25.0%)
Asthma	2644 (11.7%)	9899 (10.2%)
COPD	1594 (7.1%)	7255 (7.5%)
Interstitial Lung Disease	24 (0.1%)	129 (0.1%)
Chronic Kidney Disease	3135 (13.9%)	16633 (17.1%)
Any active Malignancy	3968 (17.6%)	17572 (18.1%)
Dementia (all types)	535 (2.4%)	3413 (3.5%)
**English Indices of deprivation**		
1	9448 (42.0%)	38534 (39.6%)
2	4638 (20.6%)	19841 (20.4%)
3	3888 (17.3%)	16630 (17.1%)
4	2200 (9.8%)	9432 (9.7%)
5	1644 (7.3%)	6914 (7.1%)
Missing	697 (3.1%)	5975 (6.1%)
Regained R2R criteria during stay? (n, %)	N/A	58609 (60.2%)
**Reason for regaining R2R criteria?**	N/A	
ICU		1727 (1.8%)
TAP surgery (72h)		263 (0.3%)
Lower limb surgery (24h)		95 (0.1%)
Invasive surgery (24h)		579 (0.6%)
Acute dependency level (48h)		7452 (7.7%)
NEWS >3 (24h)		20605 (21.2%)
O2 treatment (24h)		5111 (5.3%)

For peer review only

Intravenous fluids or treatments (24 hours, > tds)		27069 (27.8%)
GCS < or + 12 (24h)		183 (0.2%)
EOL care (24h)		165 (0.2%)
Increased dependency (48h)		10290 (10.6%)

Table S4. Demographics of patients not meeting R2R criteria on presentation to QEHB in the censor period. Legend. Data is number (percentage) of patients in a bed at 00:00 who either were or were not discharged in the subsequent twenty-four hours after eR2R assessment. Ethnicity was self-reported. Medical conditions were physician confirmed and checked against admission and linked primary care notes. English Indices of deprivation were calculated using postcode.

For peer review only

Online Supplement Figures

A

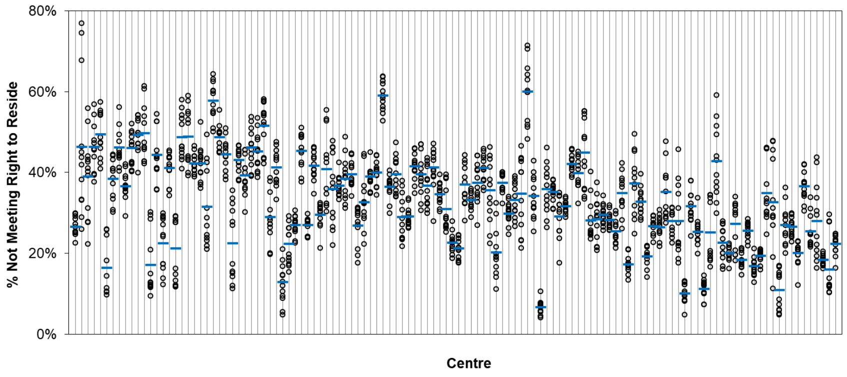

B

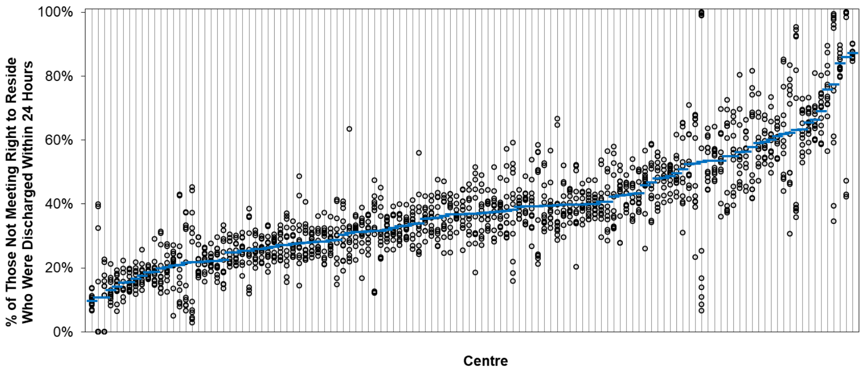

Figure S1. The proportions of patients with no right to reside (A), and proportions of these that were discharged within 24 hours (B). Analysis by week.

Legend: The proportions of patients not meeting the R2R, and the proportions of these patients that were discharged within 24 hours were extracted from daily reports for each national NHS centre. The weekly mean of each centres values was calculated for each of twelve weeks analysed and plotted as a circle. The mean across the twelve weeks analysed for each centre is plotted as a horizontal line. Centres are arranged in ascending order of the period mean proportion of patients without R2R discharged within 24 hours.

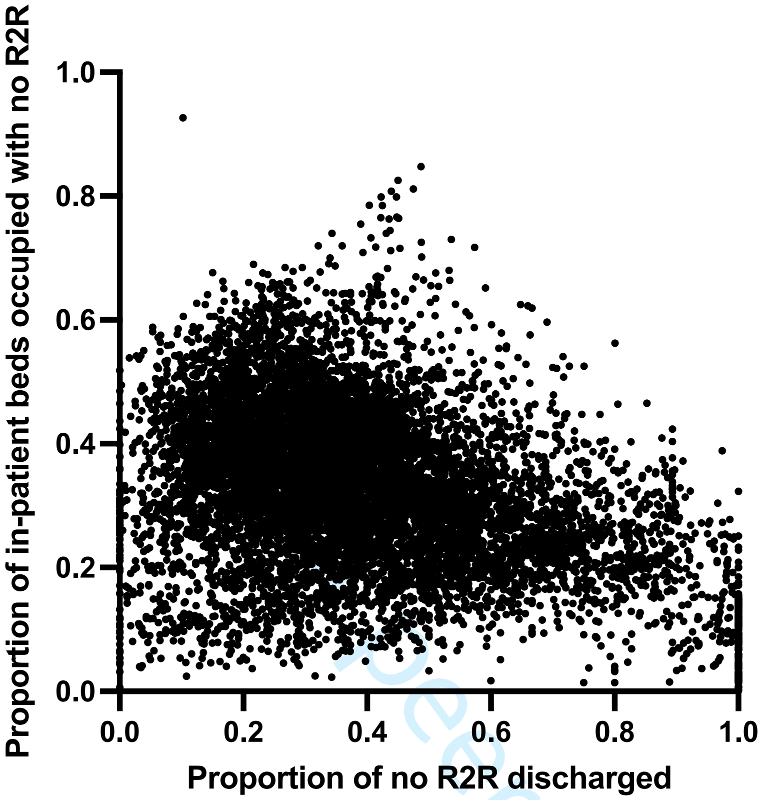

Figure S2. The proportions of patients with no R2R and of that group the proportion discharged over the next 24 hours

Legend: The proportions of patients not meeting the R2R, and of that group the proportion of patients discharged within 24 hours, reported to SDCS from 29 Nov 2021 – 20 Feb 2022 across 121 centres. Each dot represents result for a single centre-day. The two metrics were associated (slope = -0.21, p<0.0001) but the correlation was low (R^2=0.12).

For peer review only

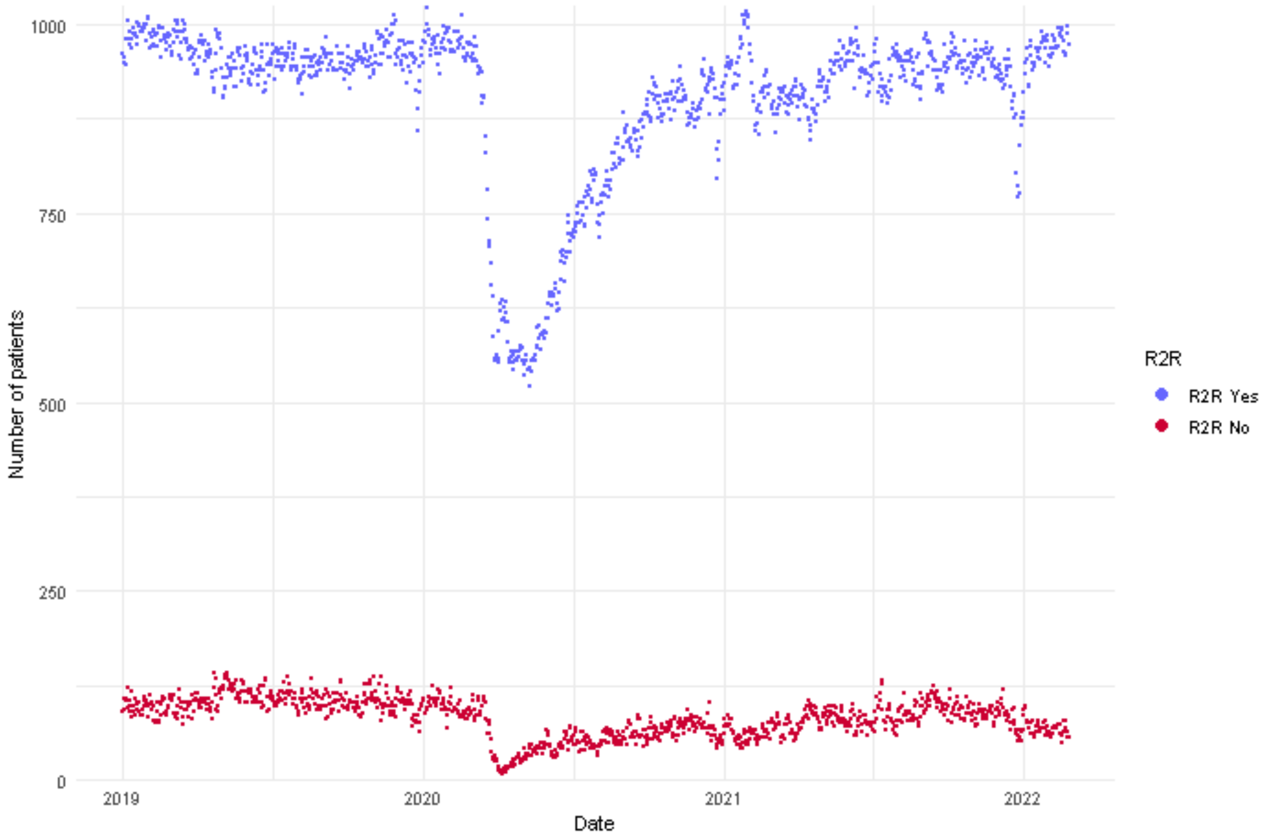

Figure S3. The number of patients meeting or not meeting eR2Rab criteria 01 Jan 2019 - 31 Dec 2021

Legend: Number of patients with (red dot) or without (blue dot) eR2Rab at 00:00 on each day of 2019-2021. The first COVID-19 admission to QEHB occurred on 1st March 2020. The first wave of the pandemic was associated with significant changes resulting in reduced bed occupancy and the majority of admitted patients had a diagnosis of COVID-19

review only

STROBE Statement—checklist of items that should be included in reports of observational studies

	Item No	Recommendation	Page
**Title and abstract**	1	(*a*) Indicate the study's design with a commonly used term in the title or the abstract	1
		(*b*) Provide in the abstract an informative and balanced summary of what was done and what was found	2
**Introduction**			
Background/rationale	2	Explain the scientific background and rationale for the investigation being reported	3
Objectives	3	State specific objectives, including any prespecified hypotheses	4
**Methods**			
Study design	4	Present key elements of study design early in the paper	6
Setting	5	Describe the setting, locations, and relevant dates, including periods of recruitment, exposure, follow-up, and data collection.	6
Participants	6	(*a*) *Cohort study*—Give the eligibility criteria, and the sources and methods of selection of participants. Describe methods of follow-up<br>*Case-control study*—Give the eligibility criteria, and the sources and methods of case ascertainment and control selection. Give the rationale for the choice of cases and controls<br>*Cross-sectional study*—Give the eligibility criteria, and the sources and methods of selection of participants	6
Variables	7	Clearly define all outcomes, exposures, predictors, potential confounders, and effect modifiers. Give diagnostic criteria, if applicable	6
Data sources/ measurement	8*	For each variable of interest, give sources of data and details of methods of assessment (measurement). Describe comparability of assessment methods if there is more than one group	6
Bias	9	Describe any efforts to address potential sources of bias	6
Study size	10	Explain how the study size was arrived at	6
Quantitative variables	11	Explain how quantitative variables were handled in the analyses.	6
Statistical methods	12	(*a*) Describe all statistical methods, including those used to control for confounding	6
		(*b*) Describe any methods used to examine subgroups and interactions	6
		(*c*) Explain how missing data were addressed	6
		(*d*) *Cohort study*—If applicable, explain how loss to follow-up was addressed<br>*Case-control study*—If applicable, explain how matching of cases and controls was addressed<br>*Cross-sectional study*—If applicable, describe analytical methods taking account of sampling strategy	6

For peer review only - http://bmjopen.bmj.com/site/about/guidelines.xhtml
For peer review only

**Results**			
Participants	13*	(a) Report numbers of individuals at each stage of study—eg numbers potentially eligible, examined for eligibility, confirmed eligible, included in the study, completing follow-up, and analysed.	7
		(b) Give reasons for non-participation at each stage	7
		(c) Consider use of a flow diagram **Online supplement**	
Descriptive data	14*	(a) Give characteristics of study participants (eg demographic, clinical, social) and information on exposures and potential confounders.	9
		(b) Indicate number of participants with missing data for each variable of interest	9
		(c) *Cohort study*—Summarise follow-up time (eg, average and total amount). N/A	
Outcome data	15*	*Cohort study*—Report numbers of outcome events or summary measures over time. N/A	
		Case-control study—Report numbers in each exposure category, or summary measures of exposure. N/A	8-10
		Cross-sectional study—Report numbers of outcome events or summary measures	
Main results	16	(*a*) Give unadjusted estimates and, if applicable, confounder-adjusted estimates and their precision (eg, 95% confidence interval). Make clear which confounders were adjusted for and why they were included	8-12
		(*b*) Report category boundaries when continuous variables were categorized	8-12
		(*c*) If relevant, consider translating estimates of relative risk into absolute risk for a meaningful time period.	8-12
Other analyses	17	Report other analyses done—eg analyses of subgroups and interactions, and sensitivity analyses	8-12
**Discussion**			
Key results	18	Summarise key results with reference to study objectives.	13-14
Limitations	19	Discuss limitations of the study, sources of potential bias or imprecision. Discuss both direction and magnitude of any potential bias	13-14
Interpretation	20	Give a cautious overall interpretation of results considering objectives, limitations, multiplicity of analyses, results from similar studies, and other relevant evidence. 13-14	
Generalisability	21	Discuss the generalisability (external validity) of the study results 14	
**Other information**			
Funding	22	Give the source of funding and the role of the funders for the present study and, if applicable, for the original study on which the present article is based. 15	

For peer review only - http://bmjopen.bmj.com/site/about/guidelines.xhtml

BMJ Open

Variability and performance of NHS England's 'Reason to Reside' criteria in predicting hospital discharge in acute hospitals in England: a retrospective, observational cohort study

Journal:	*BMJ Open*
Manuscript ID	bmjopen-2022-065862.R1
Article Type:	Original research
Date Submitted by the Author:	05-Dec-2022

Complete List of Authors:	Sapey, Elizabeth; University of Birmingham, PIONEER Data Hub; University Hospitals Birmingham NHS Foundation Trust, Acute Medicine<br>Gallier, Suzy; University Hospitals Birmingham NHS Foundation Trust<br>Evison, Felicity; University Hospitals Birmingham NHS Foundation Trust, Department of Informatics<br>McNulty, David; University Hospitals Birmingham NHS Foundation Trust, Informatics<br>Reeves, Katherine; University Hospitals Birmingham NHS Foundation Trust, Health Informatics<br>Ball, Simon; University Hospitals Birmingham NHS Foundation Trust

**Primary Subject Heading**:	Health policy
Secondary Subject Heading:	Evidence based practice, Health services research
Keywords:	Information management < BIOTECHNOLOGY & BIOINFORMATICS, Health policy < HEALTH SERVICES ADMINISTRATION & MANAGEMENT, Quality in health care < HEALTH SERVICES ADMINISTRATION & MANAGEMENT

SCHOLARONE™ Manuscripts

I, the Submitting Author has the right to grant and does grant on behalf of all authors of the Work (as defined in the below author licence), an exclusive licence and/or a non-exclusive licence for contributions from authors who are: i) UK Crown employees; ii) where BMJ has agreed a CC-BY licence shall apply, and/or iii) in accordance with the terms applicable for US Federal Government officers or employees acting as part of their official duties; on a worldwide, perpetual, irrevocable, royalty-free basis to BMJ Publishing Group Ltd ("BMJ") its licensees and where the relevant Journal is co-owned by BMJ to the co-owners of the Journal, to publish the Work in this journal and any other BMJ products and to exploit all rights, as set out in our licence.

The Submitting Author accepts and understands that any supply made under these terms is made by BMJ to the Submitting Author unless you are acting as an employee on behalf of your employer or a postgraduate student of an affiliated institution which is paying any applicable article publishing charge ("APC") for Open Access articles. Where the Submitting Author wishes to make the Work available on an Open Access basis (and intends to pay the relevant APC), the terms of reuse of such Open Access shall be governed by a Creative Commons licence – details of these licences and which Creative Commons licence will apply to this Work are set out in our licence referred to above.

Other than as permitted in any relevant BMJ Author's Self Archiving Policies, I confirm this Work has not been accepted for publication elsewhere, is not being considered for publication elsewhere and does not duplicate material already published. I confirm all authors consent to publication of this Work and authorise the granting of this licence.

For peer review only

Variability and performance of NHS England's 'Reason to Reside' criteria in predicting hospital discharge in acute hospitals in England: a retrospective, observational cohort study

Elizabeth Sapey[1,3], Suzy Gallier[2,3], Felicity Evison[4], David McNulty[5], Katherine Reeves[6], Simon Ball[7]

1a. PIONEER, HDRUK Health Data Hub in Acute Care, Birmingham, UK. University of Birmingham, Birmingham, UK. ORCiD ID: 0000-0003-3454-5482. e.sapey@bham.ac.uk

1b. Acute Medicine, University Hospitals Birmingham NHS Foundation Trust, Edgbaston, Birmingham, UK.

2. University Hospitals Birmingham NHS Foundation Trust, Edgbaston, Birmingham, UK. Suzy.Gallier@UHB.nhs.uk

3. Joint first authors.

4. University Hospitals Birmingham NHS Foundation Trust, Edgbaston, Birmingham, UK. Felicity.Evison@UHB.nhs.uk

5. University Hospitals Birmingham NHS Foundation Trust, Edgbaston, Birmingham, UK. David.McNulty@UHB.nhs.uk

6. University Hospitals Birmingham NHS Foundation Trust, Edgbaston, Birmingham, UK. Katherine.Reeves@UHB.nhs.uk

7a. Health Data Research UK (HDR-UK) Midland's Physical Site. University of Birmingham, Edgbaston, Birmingham, UK. Institute of Immunology and Immunotherapy, University of Birmingham, Birmingham, UK.

7b Chief Medical Officer, University Hospitals Birmingham NHS Foundation Trust, Edgbaston, Birmingham, UK. Simon.Ball@uhb.nhs.uk

Correspondence to:

Elizabeth Sapey

University of Birmingham, Edgbaston, Birmingham, UK, B15 2GW.

Institute of Inflammation and Ageing, University of Birmingham, Birmingham, UK.

ORCiD ID: 0000-0003-3454-5482.

Email: e.sapey@bham.ac.uk

Tel: 00 44 121 246 2000

For peer review only

Keywords

Health quality, information management, health policy, discharge planning, healthcare decision making

Word count:

Abstract: 290

Main article: 2904

For peer review only

Abstract

Objectives: NHS England (NHSE) advocates 'Reason to Reside' (R2R) criteria to support discharge planning. The proportion of patients without R2R and their rate of discharge are reported daily by acute hospitals in England. R2R has no inter-operable standardised data model (SDM) and its performance has not been validated. We aimed to understand the degree of inter- and intra-centre variation in R2R related metrics reported to NHSE, define a SDM implemented within a single centre Electronic Health Record to generate an eR2R, and evaluate its performance in predicting subsequent discharge.

Design: Retrospective observational cohort study using routinely collected health data.

Setting: 122 NHS Trusts in England for national reporting and an acute hospital in England for local reporting.

Participants: 6,602,706 patient-days were analysed using 3 months national data and 1,039,592 patient-days, using 3 years single centre data.

Main outcome measures: Variability in R2R related metrics reported to NHSE. Performance of eR2R in predicting discharge within 24 hours.

Results: There were high levels of intra and inter-centre variability in R2R related metrics ($p<0.0001$), but not in eR2R. Informedness of eR2R for discharge within 24 hours was low (J-statistic 0.09 – 0.12 across three consecutive years). In those remaining in hospital without eR2R, 61.2% met eR2R criteria on subsequent days (76% within 24 hours), most commonly due to increased NEWS2 (21.9%) or intravenous therapy administration (32.8%).

Conclusions: Reported R2R metrics are highly variable between and within acute Trusts in England. Although case-mix or community care provision may account for some variability, the absence of a SDM prevents standardised reporting. Following the development of a SDM in one acute Trust, the variability reduced. However, the performance of eR2R was poor, prone to change even when negative and unable to meaningfully contribute to discharge planning.

For peer review only

Article summary

Strengths and limitations of this study

- The intra and inter-centre variability of R2R reporting was based on national data and included > 6.6M patient bed-days.
- Standardised data model to form eR2R was based on nationally agreed criteria for each clinical question.
- All admissions > 24 hours were included for eR2R performance review, reducing bias.
- eR2R data based on one centre only, albeit one of the largest NHS Trusts nationally serving a diverse population and including >1M patient bed-days.

Introduction

In 2021 the UK Government published its policy and operating model for hospital discharge and community support within the National Health Service in England (NHSE)[1]. This policy responded to concerns about bed capacity during the COVID-19 pandemic.

A National Audit Office report recognised the potential to release acute hospital beds in 2016, finding that older patients no longer needing acute treatment accounted for 2.7 million NHS hospital bed days per year[2]. The report concluded that a lack of planning delayed discharge, recognising research which highlighted adverse outcomes during prolonged hospital stay[3, 4].

The aforementioned policy mandates using set criteria to identify in-patients in whom discharge home, or to a less acute setting, should be considered. These criteria have been referred to interchangeably, as “Reason[s] to reside” (R2R), “right to remain” or “criteria to reside” (See Table 1a). Since April 2020, NHS hospitals have been required to provide daily reports on the numbers of people leaving hospital, to where, and the reasons for those remaining in hospital. The proportion of in-patients not meeting R2R criteria, and the proportion of patients without R2R discharged that day, are also reported. These metrics are considered to be measures of organisational efficiency.

R2R appears to have emerged heuristically from the clinical experience of those involved in its development. A series of questions are posed that might prompt consideration of individual patients for discharge. However, there are no standardised data definitions, there has been no validation of R2R, no investigation of its role as a clinical decision support tool, or of its value in evaluating hospital performance. A further barrier to evaluating the performance of R2R is that there is no gold standard definition which identifies patients who could be discharged from hospital against which to compare R2R performance. This lack of a reference standard limits, but does not preclude assessment of the validity of a clinical test, provided a ‘fair’ measure of performance can be defined[5]. The set of patients actually discharged in the subsequent 24 hours is one potentially ‘fair’ test of performance of R2R.

For peer review only

Table 1. Reason to Reside (R2R)

1	Requiring ITU or HDU care
2	Requiring oxygen therapy / NIV
3	Requiring intravenous fluids
4	NEWS2 > 3 (clinical judgement required in persons with AF and/or chronic respiratory disease)
5	Diminished level of consciousness where recovery realistic
6	Acute functional impairment in excess of home/community care provision
7	Last hours of life
8	Requiring intravenous medication > bd (including analgesia)
9	Undergone lower limb surgery within 48 hours
10	Undergone throrax-abdominal/pelvic surgery within 72 hours
11	Within 24 hours of an invasive procedure? (with attendant risk of acute life-threatening deterioration)

The policy and operating model for hospital discharge and community support within the National Health Service in England states that every person on every general ward should be reviewed on a twice daily ward round to determine whether they meet R2R. If the answer to each question is 'no', the policy states that active consideration for discharge to a less acute setting must be made (1). In daily data returns, the number of patients to whom this applied were counted at a single, locally defined, time point.

For peer review only

In the current study, we show the degree of variation in R2R associated metrics reported across centres in England. Secondly, we propose precisely defined, inter-operable, data definitions corresponding to the elements of R2R. This allows for consistent, generalisable analysis. Thirdly, we evaluate the performance of R2R to predict discharge over the subsequent 24 hours.

Methods

This study used unconsented, anonymous health data and all study activity was approved by the East Midlands–Derby REC (reference: 20/EM/0158) and was supported by PIONEER, the Health Data Hub in acute care. All studies activities followed the World Medical Association's Declaration of Helsinki. The R2R criteria are as described[1] and are also provided in Table 1.

National data

National NHS England data was accessed via The UK Health Facts and Dimensions database[6] for all reporting Trusts in England. Assessment of variability in national R2R reporting included data from 29th November 2021 to 20th February 2022. Table S1 of the online supplement provides the names of the Trusts whose data are presented anonymously. Data were collected daily during the censor period for 121 centres, yielding a total of 10,164 potential data points (centre-days). For each of these, the total number of occupied and unoccupied beds, and the number of patients with no right to reside were extracted. The number of patients with no right to reside were submitted once a day by each NHS trust, based upon the local hospital interpretation of the definition provided by NHSE[1]. This required none of the criteria to be met at the time of local data collection. The numbers of patients with right to reside were then calculated by subtracting the number with no right to reside from the total number of occupied beds on that day. The number of General and Acute beds occupied in any given centre, on any given day (in-patients), was used as a surrogate for the number of patients eligible for evaluation using the R2R criteria. Review of the dataset found some missing, and potentially spurious data, which were excluded prior to analysis. This included instances where R2R data were not recorded (N=184 data points); where the total numbers of beds were either zero, missing, or clearly spurious (N=37 data points); or where there were more patients with no R2R than the total number of beds (N=3 data points). The national data are shown for the other N=121 centres, excluding UHB.

Local data

In-depth analysis of R2R criteria were performed using data from the Queen Elizabeth Hospital Birmingham (QEHB). QEHB is a National Health Service (NHS), urban, adult, acute hospital in England which in 2019 had 1269 beds including 80 level 2/3 intensive care (ICU) beds, an Emergency Department that assesses >300 patients per day, and a mixed secondary and tertiary practice that includes all major adult specialities except for obstetrics and gynaecology. The electronic healthcare record (EHR) at QEHB (PICS, Birmingham Systems) contains time-stamped, structured records that include demography, location, admission and discharge, co-morbidities, physiological measurements supporting NEWS2 and Glasgow Coma Scale, operation noting, prescribing and investigations.

The R2R criteria in Table 1 were mapped to computable definitions derived from the EHR (See Table 2), to generate an electronic R2R (eR2R). The OPCS Classification of Interventions and Procedures codes mapped to criterion 9-11 are described in Table S2 of the online supplement. The concept 'acute functional impairment in excess of home/community care provision', had no direct correlate. Safer Nursing Care Tool (SNCT) levels of care were however available[7]. SNCT level 2 and 3 correspond closely with the requirement for HDU or ICU[8]. Level

For peer review only - http://bmjopen.bmj.com/site/about/guidelines.xhtml

1a identifies patients requiring enhanced nursing reflecting acuity of illness and Level 1b identifies a group with increased nursing dependency. Level 1b is likely to include those who would and would not be considered to require ongoing care in acute hospital. SNCT level 1 was included in the definition of eR2R in two ways, including (eR2Rab) and excluding (eR2Ra) level 1b, to determine if this affected performance.

Table 2. Data definitions used to operationalise R2R for EHR

	Flag if…	R2R criterion number
On ITU HDU	listed as being in ITU or HDU ward	1
SNCT Level ≥ 2	Most recent SNCT level in previous 48 hours ≥ 2	1
SNCT Level 1a	Most recent SNCT level in previous 48 hours = 1a	6
SNCT Level 1b	Most recent SNCT level in previous 48 hours = 1b	6
Oxygen therapy/ NIV	oxygen administration or NIV<br>documented in observation chart within previous 24 hours	2
Intravenous fluids	iv fluid administration initiated in previous 24 hours or<br>variable rate insulin infusion administered in previous 24 hours	3
NEWS2	if NEWS2 > 3 within last 24 hrs	4
Diminished consciousness	Glasgow Coma Scale value ≤ 12 in last 24 hours	5
Last hours of life	comfort observation completed current<br>OR<br>End of Life medication bundle administered within last 24 hours	7
Intravenous prescription ≥ tds current (regular not prn)	IV medication prescribed within last 24 hours and frequency ≥ ≥ 3 times per day for regular medication only	8
Intravenous medication administration ≥ tds within 24 hrs	IV medication administered ≥ 3 times within last 24 hours	8
Lower limb surgery within 48hrs	Procedure with relevant OPCS codes in previous 48 hours	9
Thorax-abdominal-pelvic surgery with 72hrs	Procedure with OPCS relevant codes in previous 72 hours	10
Invasive procedure within 24hrs	Procedure with OPCS relevant codes in previous 24 hours	11

The table describes the data definitions used and the R2R criteria they map to. ITU = intensive care. HDU = High dependency unit. SNCT = Safer Nursing Care Tool. NEWS2 = National Early Warning Score 2. tds = thrice daily. OPCS = OPCS Classification of Interventions and Procedures code which is used to identify the coded clinical entry. All OPCS codes used to identify procedures are listed in Table S2 of the online supplement.

For peer review only

The primary analysis of eR2R was for patients who had been in hospital for more than twenty-four hours at midnight. Discharge over the course of the subsequent twenty-four hours was evaluated. Secondary analyses were undertaken for the set of patients in a bed at 08:00 and at 16.00 to define any change in eR2R performance in these different cross sections of the in-patient population. Three calendar years were analysed separately, to assess the effects of the COVID19 pandemic.

Statistics

Initially, daily numbers of patients with R2R quantified both as absolute numbers and a proportion of the total number of beds, were plotted for national centres and used to calculate between-centre and within-centre variation. These data are analysed as beds occupied at the specified time of day, where the bed inherits the demographics, comorbidities, and other qualities of the occupying patient. This represents the in-patient population in cross-section.

For the local analysis of eR2R: the term patient-day was used to refer to a bed with the qualities of the occupying patient at the time of the analysis. The in-patient population is described as means of patient-days thereby representing a cross-section of the group. The performance of eR2R as a predictor of remaining in hospital (or absence of eR2R as a predictor of discharge) was reported as a True Positive Rate (TPR) and True Negative Rate (TNR), Positive Predictive Value (PPV), Negative Predictive Value (NPV) and Youden's J statistic (TPR+TNR-1), where positive is remains in hospital and negative is discharge from hospital within 24 hours.

Normally distributed variables are reported as arithmetic means ± standard deviations, with medians and ranges used otherwise. Between-centre variation was assessed by ANOVA. This included a model accounting for day of the week as a fixed effect and the centre as a random effect. All analyses were performed using IBM SPSS 22 (IBM Corp. Armonk, NY), with $p<0.05$ deemed to be indicative of statistical significance throughout

Patient and public involvement

The research question and topic were agreed following patient/public discussion groups about NHSE discharge policies. Patients/public reviewed the data fields included in the study, with the PIONEER Data Trust Committee providing support for the project (a group of patient/public members who review studies using health data[9]). A patient/public group have reviewed the results and have written a lay summary for study dissemination to patient groups

Results

R2R reporting in England, Nov 20-Feb 21

For peer review only

Across 10,164 available centre-days, accounting for 6,602,706 patient-days, the number of patients reported without R2R as a proportion of in-patients, varied significantly between centres ($p<0.0001$). Individual centre means ranged from 6.7% ± 2.5% to 59.9% ± 13.8% (Figure 1a). There was also marked within-centre variation (Figure 1a), with coefficients of variation (CV) ranging from 8.2% up to 59.3%. Of patients not meeting R2R criteria, the proportion discharged over the following 24 hours, varied significantly between centres ($p<0.0001$). Individual centre means ranged from 14.0% ± 7.4% to 85.8% ± 25.2% (Figure 1b). There was also marked within centre variation, with coefficients of variation ranging from 6.4% up to 83.2%. These data are shown as median and IQR in Figure S1a and S1b of the online supplement). The proportion of patients without R2R and the proportion of that group discharged within 24 hours, were only weakly correlated (R^2=0.12; Figure S2 of the online supplement).

Performance of eR2R at QEHB

Standardised definitions corresponding to the elements of R2R (Table 2) were used to analyse data from QEHB, on 1,214,480 in-patient days, between 01 Jan 2019 – 31 Dec 2021. The demographic and clinical details of that population are summarised in Table 3 which also shows that those meeting the definition of eR2Rab were older and more likely to have one or more co-morbidities than those who did not. Variation in the daily number of patients with or without an eR2R is shown in Figure S3 of the online supplement.

Table 3. Demographics of patients meeting and not meeting R2R criteria on presentation to QEHB in the censor period

	All QEHB patient days	Meeting eR2Rab	Not meeting eR2Rab
N	1039592	919751 (88.5%)	119841 (11.5%)
**Age in years***: median (IQR)	68 (53-80)	69 (54-81)	63 (48-76)
**Sex* (n, %)**			
Female	488120 (47.0%)	434418 (47.2%)	53702 (44.8%)
Male	546061 (52.5%)	484816 (52.7%)	61245 (51.1%)
Not recorded	5411 (0.5%)	517 (0.1%)	4894 (4.1%)
**Self-reported ethnicity* (n, %)**			
White	784528 (75.5%)	698573 (76.0%)	85955 (71.7%)
Mixed/ Multiple	12983 (1.2%)	11023 (1.2%)	1960 (1.6%)
South Asian/ Asian British	114049 (11.0%)	98903 (10.8%)	15146 (12.6%)
Black/ African/ Caribbean/ Black British	51122 (4.9%)	43991 (4.8%)	7131 (6.0%)
Other ethnic group	19475 (1.9%)	16623 (1.8%)	2852 (2.4%)
Not known	57435 (5.5%)	50638 (5.5%)	6797 (5.7%)
**Co-morbidity count* (n, %)**			
None	196121 (18.9%)	164704 (17.9%)	31417 (26.2%)
1-2	474922 (45.7%)	423200 (46.0%)	51722 (43.2%)
3 or more	368549 (35.5%)	331847 (36.1%)	36702 (30.6%)
**Morbidities (n, %)**			

Hypertension*	492160 (47.3%)	439930 (47.8%)	52230 (43.6%)
Cerebrovascular disease*	159316 (15.3%)	147676 (16.1%)	11640 (9.7%)
Atrial fibrillation*	224501 (21.6%)	204458 (22.2%)	20043 (16.7%)
Ischaemic heart disease, angina, myocardial infarct*	198480 (19.1%)	173708 (18.9%)	24772 (20.7%)
Diabetes (type 1 and 2)*	271505 (26.1%)	242328 (26.3%)	29177 (24.3%)
Asthma*	103679 (10.0%)	91136 (9.9%)	12543 (10.5%)
COPD*	112731 (10.8%)	103882 (11.3%)	8849 (7.4%)
Interstitial Lung Disease*	2533 (0.2%)	2380 (0.3%)	153 (0.1%)
Chronic Kidney Disease*	198052 (19.1%)	178284 (19.4%)	19768 (16.5%)
Any active Malignancy *	215959 (20.8%)	194419 (21.1%)	21540 (18.0%)
Dementia (all types)*	65272 (6.3%)	61324 (6.7%)	3948 (3.3%)
**English Indices of deprivation**			
1	430114 (41.4%)	382132 (41.5%)	47982 (40.0%)
2	222478 (21.4%)	197999 (21.5%)	24479 (20.4%)
3	178565 (17.2%)	158047 (17.2%)	20518 (17.1%)
4	107747 (10.4%)	96115 (10.5%)	11632 (9.7%)
5	75854 (7.3%)	67296 (7.3%)	8558 (7.1%)
Not recorded	24834 (2.4%)	18162 (2.0%)	6672 (5.6%)
**Care escalation to ITU (n, %)**	101017 (9.7%)	93080 (10.1%)	7937 (6.6%)

Data is number (percentage) of patients in a bed at 00:00. Ethnicity was self-reported. Medical conditions were physician confirmed and checked against admission and linked primary care notes. English Indices of deprivation were calculated using postcode. *Significant difference between meeting and not meeting eR2Rab ($p<0.05$ in univariate analysis).

Criteria contributing to eR2R

Given the potential for the COVID19 pandemic to affect R2R, calendar years were analysed separately. The number of patients meeting any given eR2R criterion are shown in Table 4a. The progressive contribution of different elements of the definition of eR2R assessed daily in a modified Consort table, are summarised in Table 4b. The proportion of patients not meeting eR2R criteria exhibited relatively little day to day variation in 2019 (eR2Rab, CV = 11.2%; eR2Ra, CV = 6.3%), although somewhat higher in the context of case mix variation consequent upon peaks of patients admitted with COVID-19 in 2020 (eR2Rab, CV = 23.3%; eR2Ra, CV = 14.4%) and 2021 (eR2Rab, CV=17.1%; eR2Ra, CV = 9.9%). The criteria contributing most to eR2R status included acuity level (NEWS2 >3), SNCT level nursing requirement, being on intensive care and requiring intravenous medications or fluids.

For peer review only

Table 4a. The number (percentage) of patient-days on which each eR2R data definition was met

Year	2019	2020	2021
**Criterion**	n (%)	n (%)	n (%)
ICU	22899 (6.1)	20326 (6.7)	21305
TAP surgery 72Hrs	3783 (1.0)	3010 (1.0)	3974
Lower limb surgery 48Hrs	285 (0.1)	252 (0.1)	221 (0.1)
Invasive surgery 24Hrs	1861 (0.5)	1613 (0.5)	1988 (0.6)
NEWS2 > 3 24hrs	93501 (24.8)	85123 (27.9)	97722 (27.3)
O2 Treatment 24Hrs	77949 (20.7)	69355 (22.7)	77202 (21.6)
Insulin Infusion 24Hrs	10951 (2.9)	10860 (3.6)	12496 (3.5)
IV Fluids 24Hrs	79802 (21.2)	71376 (23.4)	80246 (22.4)
IV medication administered in last 24hrs >= tds	95034 (25.2)	81174 (26.6)	91573 (25.6)
IV medication prescribed in last 24Hrs >= tds	21543 (5.7)	17866 (5.9)	19249 (5.4)
SNCT Dependency 1a, 2, 3	99139 (26.3)	72226 (23.7)	88832 (54.8)
COMA Score <=12 in last 24Hrs	6594 (1.8)	6448 (2.1)	6664 (1.9)
End of Life care definition met in last 24Hrs	5359 (1.4)	4747 (1.6)	5075 (1.4)
SNCT Dependency 1b	172659 (45.8)	160380 (52.5)	179527 (50.2)
Total number of patient days	376684	305254	357654

The number (percentage) of patient days on which each eR2R definition was met. The population was in-patients at 24.00 with length of stay ≥ 24 hours.

Table 4b: A phased analysis undertaken for each day and presented as a modified Consort Diagram

Year	2019	2020	2021
**Criterion**	Mean % (SD)	Mean % (SD)	Mean % (SD)
ICU	6.1% (0.44)	7.1% (3.10)	6.0% (2.16)
TAP surgery 72Hrs	0.7% (0.35)	0.7% (0.37)	0.8% (0.45)
Lower limb surgery 48Hrs	0.1% (0.07)	0.1% (0.11)	0.1% (0.08)
Invasive surgery 24Hrs	0.2% (0.15)	0.2% (0.18)	0.2% (0.15)
NEWS2 > 3 24hrs	24.2% (2.28)	27.5% (3.82)	26.6% (3.64)
O2 Treatment 24Hrs	4.0% (0.61)	3.9% (0.72)	3.6% (0.68)

For peer review only

Insulin Infusion 24Hrs	0.5% (0.24)	0.6% (0.28)	0.5% (0.23)
IV Fluids 24Hrs	8.8% (1.09)	9.5% (1.37)	9.6% (1.24)
IV Medication Admin 24Hrs >= tds	7.7% (1.05)	7.4% (1.29)	7.5% (1.17)
IV Medication Prescribed 24Hrs	0.7% (0.28)	0.6% (0.29)	0.6% (0.27)
SNCT Dependency 1a, 2, 3	8.8% (1.42)	6.7% (1.21)	7.8% (1.12)
COMA Score <=12 24Hrs	0.0% (0.05)	0.0% (0.08)	0.0% (0.06)
End of Life 24Hrs	0.5% (0.24)	0.4% (0.27)	0.4% (0.19)
SNCT Dependency 1b	24.5% (1.88)	25.5% (3.53)	25.3% (2.59)
No eR2Rab total	13.3% (1.50)	9.8% (2.29)	10.9% (1.87)
No eR2Ra total	37.8% (2.38)	35.3% (5.08)	36.2% (3.60)

The progressive contribution of each element to the definition of eR2R was calculated as proportion of the whole population. These were aggregated by calendar year. The order of the phased analysis was determined by the researchers to be that which was most informative, and which placed objective definitions earlier. SNCT dependency is a global nursing assessment and therefore was placed last.

Informedness of eR2R for discharge in the next 24 hours

For the outcome discharge (remain -) / no discharge (remain +) within 24 hours, across the 3 different years, the eR2Ra TPR lay between 0.63 and 0.65, TNR between 0.46 and 0.47, the PPV was 0.91 and NPV between 0.12 and 0.15; the eR2Rab TPR lay between 0.88 and 0.91, TNR between 0.18 and 0.24, the PPV between 0.90 and 0.91 and NPV between 0.18 and 0.20 (Table 5). The J statistic for both definitions lay between 0.09-0.12. In secondary analyses based upon the in-patient population at 08.00 and at 16.00 the J-statistic ranged between 0.10-0.14 and 0.10-0.15 respectively (Tables S3a and S3b of the online supplement).

Table 5. Contingency tables showing the number of patients meeting criteria for (A) eR2Ra and (B) eR2ab

A.

	**2019**	**Remain**		
		Yes (+)	No (-)	Total
**eR2Ra**	Yes (+)	213,382	20,845	234,227
	No (-)	124,874	17,583	142,457
	Total	338,256	38,428	376,684
	**2020**	**Remain**		

B.

	**2019**	**Remain**		
		Yes (+)	No (-)	Total
**eR2Rab**	Yes (+)	297,172	29,372	326,544
	No (-)	41,084	9,056	50,140
	Total	338,256	38,428	376,684
	**2020**	**Remain**		

		Yes (+)	No (-)	Total
**eR2Ra**	Yes (+)	177,065	18,292	195,357
	No (-)	93,947	15,950	109,897
	Total	271,012	34,242	305,254
	**2021**	**Remain**		
		Yes (+)	No (-)	Total
**eR2Ra**	Yes (+)	208,068	20,084	228,152
	No (-)	112,007	17,495	129,502
	Total	320,075	37,579	357,654

		Yes (+)	No (-)	Total
**eR2Rab**	Yes (+)	246,461	28,026	274,487
	No (-)	24,551	6,216	30,767
	Total	271,012	34,242	305,254
	**2021**	**Remain**		
		Yes (+)	No (-)	Total
**eR2Rab**	Yes (+)	288,384	30,336	318,720
	No (-)	31,691	7,243	38,934
	Total	320,075	37,579	357,654

The tables show numbers of patients meeting R2R criteria and the corresponding number of patients who remain in hospital over the next 24 hours or do not (were discharged), for the in-patient population at 00:00. For eR2Ra, the TPR varied between 0.62-0.65 and TNR 0.46-0.51, across 3 different years and 3 different time points. For eR2Rab, the TPR varied between 0.87-0.91 and TNR 0.18-0.25, across 3 different years and 3 different time points. Table S3 of the online supplement shows the same data for the in-patient population at 16:00. See Table S4 of the online supplement for all sensitivity and specificity analysis.

In-patients not meeting eR2R

The demographic and clinical details of patient who did not meet the eR2Rab definition, stratified by discharge in the subsequent 24 hours are shown in Table S5 of the online supplement. For patient-days on which discharge occurred within 24 hours, there was significantly higher representation of those with no documented co-morbidities 29.2% vs 24.0% (p<0.0001). In those that remained in hospital, 61.2% met eR2R criteria on subsequent days (76% within the next 24 hours). Of all those that remained, 21.9% acquired a NEWS2 > 3, 32.8% received iv fluids or drugs > 3 times / day and 1.9% were admitted to ICU.

Discussion

Assessment of an individual patient's R2R has been promoted as a tool to improve the identification of those who could be discharged from acute hospitals in England. The proportion of in-patients with R2R and their rate of discharge has then been used to evaluate the operational efficiency of acute hospitals and their adjacent health and social care system[1, 10]. This paper presents findings to suggest that as currently constituted, R2R is of limited value for these purposes.

The high levels of variation in R2R related metrics, within and between centres in England, has been attributed to variation in case mix and operational efficiency[11]. However, such extremes of variation are not observed in other metrics that use established data standards. Furthermore, the proportion of patients not meeting R2R

For peer review only

criteria correlates poorly with their rate of discharge over the subsequent 24 hours, whereas one might anticipate that such closely related measures of operational efficiency would reflect one another. These findings are most obviously accounted for by the fact that R2R does not constitute a semantic data model. It is therefore susceptible to differing interpretation by individuals and centres. This applies to all the concepts described by R2R, but most obviously those that are necessarily subjective, such as 'acute functional impairment in excess of home/community care provision' and 'diminished level of consciousness where recovery is realistic'[12, 13].

We therefore developed machine readable data definitions corresponding to each concept, allowing consistent analysis of R2R at scale, using data derived from the EHR in our centre. The SNCT is a global nursing assessment of acuity and dependency that was developed to guide workforce deployment. It is regularly recorded within the EHR at our centre. Because Level 1b describes a group of patients who are highly dependent upon nursing care for daily activities, this was mapped onto the R2R concept 'acute functional impairment in excess of home/community care provision'. However, since the definition of level 1b could include a group of patients suitable for discharge to a less acute setting, two definitions or eR2R were tested, with and without SNCT 1b. Our analysis is therefore likely to represent two extremes of inclusion of patients with acute functional impairment.

Within centre variation in eR2R was low, consistent with it minimising individual interpretation of each data element. eR2R was a poor predictor of discharge within 24 hours[14]. Youden's Index was consistently <0.15 across 3 calendar years, 3 different times of day and two eR2R definitions. For a dichotomous test such as eR2R, a Youden's Index >0.50 is generally considered the empirical benchmark for a test to support clinical decision making[15]. eR2R is therefore unsuited to the provision of clinical decision support tool for discharge. It does not define a sub-population on which to assess discharge performance[16]. The limitations of R2R are not entirely surprising, given the need to interpret concepts that are not semantically defined. Although addressed by eR2R, it nevertheless remains a simple series of binary responses to questions that have not been validated for the purpose of discharge prediction. For example, NEWS2 was validated as an acuity score to quantify physiological instability on initial presentation to hospital[17]. It was not developed and has not been validated, as a triage tool to assess fitness to leave hospital, at any threshold.

Importantly, more than half of those who remain in hospital without eR2R, subsequently acquired eR2R. This group of patients were older and had multiple long-term health conditions, suggesting that there were clinical grounds for that decision, albeit undefined. This sub-population requires further study.

There are limitations to our analysis. The eR2R was assessed in only one centre, albeit one that serves a diverse, multi-ethnic, urban population, in which more than 1.2 million patient days were assessed. Patients admitted for < 24 hours at the time of analysis were excluded, to allow clinical decisions to be made and executed. The first day post-admission is a highly dynamic situation, with frequent clinical review; a setting in which this embodiment of clinical decision support is arguably less relevant. Another, more intrinsic problem, is that there is no gold standard by which to define all patients suitable for discharge, so that actual discharge was used as a

For peer review only

fair test when evaluating the performance of eR2R[18]. This assumes that patients actually discharged are part of a continuous population of all those who could be discharged. It is also the case that each eR2R data element could be defined in different ways, however each definition would relate to that used, so that the performance of one model would be informed by the other. For example, the 24-hour retrospective time horizon for most evaluations could be altered, but the later model would relate directly to the former.

It is important to validate and evaluate tests within their intended setting. The effects of embedding new care pathways or tools within clinical service delivery, without appropriate evaluation, are increasingly described. There is significant opportunity for unintended consequences to arise from the implementation of poorly considered clinical decision support[19], particularly when there is competition for clinical resource. This has been recently discussed for NEWS2[20], sepsis alerting and COVID-19 virtual wards[21]. R2R has been endorsed and adopted but without validation or consideration of the unintended consequences of its application. This is not to contend that a significant number of in-patients could not be discharged earlier, simply that there is no evidence that R2R can support clinical decision making. The collective limitations of R2R identified are likely to account for variation in nationally reported metrics which are difficult to explain.

Our study highlights the need for reproducible standardised data definitions to support both implementation and validation of any tool that purports to support clinical decision making. Further research should focus on building, validating and refining tools to inform clinical decisions.

*** ***

Acknowledgements

This work was supported by the PIONEER Data Hub (see www.pioneerdatahub.co.uk) and by the PIONEER patient and public advisory group and Data Trust Committee.

Transparency statement

Professor Ball (the manuscript's guarantor) affirms that the manuscript is an honest, accurate, and transparent account of the study being reported; that no important aspects of the study have been omitted; and that any discrepancies from the study as originally planned (and, if relevant, registered) have been explained.

Data availability statement

The anonymised dataset used for analysis is available upon reasonable request from the PIONEER Data Hub on submission of a data request form, see www.pioneerdatahub.co.uk for a copy of the form and processes for data access.

Competing interests

For peer review only

Felicity Evison, David McNulty, Katherine Reeves have no relevant conflicts of interest. Suzy Gallier reports grant funding from HDR-UK. Elizabeth Sapey reports grant funding from HDR UK, Innovate UK, MRC, NIHR, British Lung Foundation and Alpha 1 Foundation. Simon Ball reports funding from HDR-UK.

Contributors

Simon Ball and Elizabeth Sapey conceived the study, Suzy Gallier, Felicity Evison, David McNulty, Katherine Reeves conducted data analysis. Elizabeth Sapey and Suzy Gallier wrote the first draft of the study. All authors contributed to the study manuscript. Simon Ball is senior author and manuscript guarantor.

Ethics approval

This study used unconsented, anonymous health data and all study activity was approved by the East Midlands–Derby REC (reference: 20/EM/0158) and was supported by PIONEER, the Health Data Hub in acute care. All studies activities followed the World Medical Association's Declaration of Helsinki.

Funding

This work was funded by Health Data Research - UK (PIONEER2019).

References

1. UK Government. Hospital Discharge and Community Support: Policy and Operating Model. https://assetspublishingservicegovuk/government/uploads/system/uploads/attachment_data/file/1026672/hospital-discharge-and-community-support-policy-and-operating-model-oct-2021pdf. 2021;Date of access 13th Feb 2022.

2. National Audit Office. Discharging older patients from hospital. Report by the Comptroller and Auditor General. https://wwwnaoorguk/wp-content/uploads/2015/12/Discharging-older-patients-from-hospitalpdf. 2016;Date of access: 12th February 2022.

3. Hartley P, Costello P, Fenner R, Gibbins N, Quinn É, Kuhn I, et al. Change in skeletal muscle associated with unplanned hospital admissions in adult patients: A systematic review and meta-analysis. PloS one. 2019;14(1):e0210186-e.

4. Mudge AM, McRae P, Hubbard RE, Peel NM, Lim WK, Barnett AG, et al. Hospital-Associated Complications of Older People: A Proposed Multicomponent Outcome for Acute Care. J Am Geriatr Soc. 2019;67(2):352-6.

5. Rutjs A, Reitsma J, Coomarasamy A, Khan K, Bossuyt P. Evaluation of diagnostic tests when there is no gold standard.A review of methods. Health Technology Assessment. 2007;11:1 - 72.

6. UK Health Facts. UK Health Facts Database. https://factsanddimensionscouk/ukhf. 2022;Date of access 22nd February 2022.

7. NHS England. Safer nursing care tool. https://wwwenglandnhsuk/nursingmidwifery/safer-staffing-nursing-and-midwifery/safer-nursing-care-tool/. 2021;Date of access: 1st February 2022.

8. National Health Service. CRITICAL CARE LEVEL, The level of critical care provided during a Hospital Provider Spell. https://wwwdatadictionarynhsuk/attributes/critical_care_levelhtml. 2021;Date of access; 22nd February 2022.

9. Gallier S, Price G, Pandya H, McCarmack G, James C, Ruane B, et al. Infrastructure and operating processes of PIONEER, the HDR-UK Data Hub in Acute Care and the workings of the Data Trust Committee: a protocol paper. BMJ Health Care Inform. 2021;28(1).

10. Department of Health and Social Care. QUICK GUIDE: DISCHARGE TO ASSESS. https://wwwnhsuk/NHSEngland/keogh-review/Documents/quick-guides/Quick-Guide-discharge-to-accesspdf. 2016;Date of access: 10th February 2022.

11. Vaughan L, Bardlsey M, Bell D, Davies M, Goddard A, Imison C, et al. Models of generalist and specialist care in smaller hospitals in England: a mixed-methods study. NIHR Journals Library; 2021 Feb (Health Services and Delivery Research, No 94) Available from: https://wwwncbinlmnihgov/books/NBK568036/ doi: 103310/hsdr09040. 2021;Date of access 24th March 2022.

12. de Lange E, Verhaak P.F., van der Meer K. Prevalence, presentation and prognosis of delirium in older people in the population, at home and in long term care: a review. Geriatric Psychiatry. 2012;28(1099-1166 (Electronic)):127 - 34.

13. Morandi A, Davis D, Bellelli G, Arora RC, Caplan GA, Kamholz B, et al. The Diagnosis of Delirium Superimposed on Dementia: An Emerging Challenge. J Am Med Dir Assoc. 2017;18(1):12-8.

14. Youden WJ. Index for rating diagnostic tests. Cancer. 1950;3(0008-543X (Print)):32 - 5.

15. Hilden J, Glasziou P. Regret graphs, diagnostic uncertainty and Youden's Index. Stat Med. 1996;15(0277-6715 (Print)):969-86.

16. Schisterman EF, Faraggi D, Reiser B, Hu J. Youden Index and the optimal threshold for markers with mass at zero. Stat Med. 2008;27(2):297-315.

17. Royal College of Physicians. National early warning score (news) 2: standardising the assessment of acute-illness severity in the NHS. updated report of a working Party. https://wwwrcplondonacuk/projects/outputs/national-early-warning-score-news-2. 2017;Date of access: 14th February 2022.

18. Umemneku Chikere CM, Wilson K, Graziadio S, Vale L, Allen AJ. Diagnostic test evaluation methodology: A systematic review of methods employed to evaluate diagnostic tests in the absence of gold standard – An update. PLOS ONE. 2019;14(10):e0223832.

19. Liu X, Glocker B, McCradden MM, Ghassemi M, Denniston AK, Oakden-Rayner L. The medical algorithmic audit. The Lancet Digital Health.

20. Pankhurst T, Sapey E, Gyves H, Evison F, Gallier S, Gkoutos G, et al. Evaluation of NEWS2 response thresholds in a retrospective observational study from a UK acute hospital. BMJ Open. 2022;12(2):e054027.

21. Gallier S, Atkin C, Reddy-Kolanu V, Parekh D, Zou X, Evison F, et al. Applying a COVID Virtual Ward model, assessing patient outcomes and staff workload. Acute Med. 2021;20(4):266-75.

Figure title/legend

For peer review only

Figure 1. National reporting of R2R criteria

The proportion of patients with no R2R (Figure 1A) and of that group the proportion of patients discharged within 24 hours (Figure 1B) reported to SDCS from 29 Nov 2021 – 20 Feb 2022 across 121 centres. Each dot represents result for a single centre-day. We have ordered centres in both Figure 1 a and 1 b according to the median value of proportion of patients with R2R. (See Fig S3 for median and interquartile ranges).

For peer review only

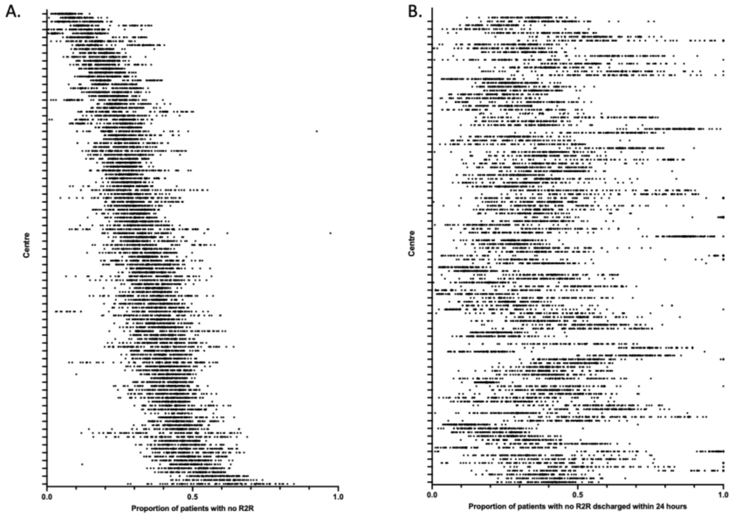

Figure 1. National reporting of R2R criteria
Legend. The proportion of patients with no R2R (Figure 1A) and of that group the proportion of patients discharged within 24 hours (Figure 1B) reported to SDCS from 29 Nov 2021 – 20 Feb 2022 across 121 centres. Each dot represents result for a single centre-day. We have ordered centres in both Figure 1 a and 1 b according to the median value of proportion of patients with R2R. (See Fig S3 for median and interquartile ranges)

338x190mm (150 x 150 DPI)

Online Supplement: The variability and performance of NHS Englands' "Reason to Reside" criteria in predicting hospital discharge in acute hospitals in England

<u>Online Tables</u>

National Trusts	
•Airedale NHS Foundation Trust	•Salisbury NHS Foundation Trust
•Ashford And St Peter's Hospitals NHS Foundation Trust	•Sandwell And West Birmingham Hospitals NHS Trust
•Barking, Havering And Redbridge University Hospitals NHS Trust	•Sheffield Teaching Hospitals NHS Foundation Trust
•Barnsley Hospital NHS Foundation Trust	•Sherwood Forest Hospitals NHS Foundation Trust
•Barts Health NHS Trust	•Somerset NHS Foundation Trust
•Bedfordshire Hospitals NHS Foundation Trust	•South Tees Hospitals NHS Foundation Trust
•Blackpool Teaching Hospitals NHS Foundation Trust	•South Tyneside And Sunderland NHS Foundation Trust
•Bolton NHS Foundation Trust	•South Warwickshire NHS Foundation Trust
•Bradford Teaching Hospitals NHS Foundation Trust	•Southport And Ormskirk Hospital NHS Trust
•Buckinghamshire Healthcare NHS Trust	•St George's University Hospitals NHS Foundation Trust
•Calderdale And Huddersfield NHS Foundation Trust	•St Helens And Knowsley Teaching Hospitals NHS Trust
•Cambridge University Hospitals NHS Foundation Trust	•Stockport NHS Foundation Trust
•Chelsea And Westminster Hospital NHS Foundation Trust	•Surrey And Sussex Healthcare NHS Trust
•Chesterfield Royal Hospital NHS Foundation Trust	•Tameside And Glossop Integrated Care NHS Foundation Trust
•Countess Of Chester Hospital NHS Foundation Trust	•The Dudley Group NHS Foundation Trust
•County Durham And Darlington NHS Foundation Trust	•The Hillingdon Hospitals NHS Foundation Trust
•Croydon Health Services NHS Trust	•The Newcastle Upon Tyne Hospitals NHS Foundation Trust
•Dartford And Gravesham NHS Trust	•South Warwickshire NHS Foundation Trust
•Doncaster And Bassetlaw Teaching Hospitals NHS Foundation Trust	•Southport And Ormskirk Hospital NHS Trust
•Dorset County Hospital NHS Foundation Trust	•St George's University Hospitals NHS Foundation Trust
•East And North Hertfordshire NHS Trust	•St Helens And Knowsley Teaching Hospitals NHS Trust
•East Cheshire NHS Trust	•Stockport NHS Foundation Trust
•East Kent Hospitals University NHS Foundation Trust	•Surrey And Sussex Healthcare NHS Trust
•East Lancashire Hospitals NHS Trust	•Tameside And Glossop Integrated Care NHS Foundation Trust
•East Suffolk And North Essex NHS Foundation Trust	•The Dudley Group NHS Foundation Trust
•East Sussex Healthcare NHS Trust	•The Hillingdon Hospitals NHS Foundation Trust
•Epsom And St Helier University Hospitals NHS Trust	•The Newcastle Upon Tyne Hospitals NHS Foundation Trust
•Frimley Health NHS Foundation Trust	•The Princess Alexandra Hospital NHS Trust
•Gateshead Health NHS Foundation Trust	•The Queen Elizabeth Hospital, King's Lynn, NHS Foundation Trust
•George Eliot Hospital NHS Trust	•The Rotherham NHS Foundation Trust
•Gloucestershire Hospitals NHS Foundation Trust	•The Royal Wolverhampton NHS Trust
•Great Western Hospitals NHS Foundation Trust	•The Shrewsbury And Telford Hospital NHS Trust
•Guy's And St Thomas' NHS Foundation Trust	•Torbay And South Devon NHS Foundation Trust
•Hampshire Hospitals NHS Foundation Trust	•United Lincolnshire Hospitals NHS Trust
•Harrogate And District NHS Foundation Trust	•University College London Hospitals NHS Foundation Trust
•Homerton University Hospital NHS Foundation Trust	
•Hull University Teaching Hospitals NHS Trust	
•Imperial College Healthcare NHS Trust	
•Isle Of Wight NHS Trust	
•James Paget University Hospitals NHS Foundation Trust	
•Kettering General Hospital NHS Foundation Trust	
•King's College Hospital NHS Foundation Trust	
•Kingston Hospital NHS Foundation Trust	
•Lancashire Teaching Hospitals NHS Foundation Trust	

For peer review only

•Leeds Teaching Hospitals NHS Trust<br>•Lewisham And Greenwich NHS Trust<br>•Liverpool University Hospitals NHS Foundation Trust<br>•London North West University Healthcare NHS Trust<br>•Maidstone And Tunbridge Wells NHS Trust<br>•Manchester University NHS Foundation Trust<br>•Medway NHS Foundation Trust<br>•Mid And South Essex NHS Foundation Trust<br>•Mid Cheshire Hospitals NHS Foundation Trust<br>•Mid Yorkshire Hospitals NHS Trust<br>•Milton Keynes University Hospital NHS Foundation Trust<br>•Norfolk And Norwich University Hospitals NHS Foundation Trust<br>•North Bristol NHS Trust<br>•North Cumbria Integrated Care NHS Foundation Trust<br>•North Middlesex University Hospital NHS Trust<br>•North Tees And Hartlepool NHS Foundation Trust<br>•North West Anglia NHS Foundation Trust<br>•Northampton General Hospital NHS Trust<br>•Northern Care Alliance NHS Foundation Trust<br>•Northern Devon Healthcare NHS Trust<br>•Northern Lincolnshire And Goole NHS Foundation Trust<br>•Northumbria Healthcare NHS Foundation Trust<br>•Nottingham University Hospitals NHS Trust<br>•Oxford University Hospitals NHS Foundation Trust<br>•Portsmouth Hospitals University National Health Service Trust<br>•Royal Berkshire NHS Foundation Trust<br>•Royal Cornwall Hospitals NHS Trust<br>•Royal Devon And Exeter NHS Foundation Trust<br>•Royal Free London NHS Foundation Trust<br>•Royal Surrey County Hospital NHS Foundation Trust<br>•Royal United Hospitals Bath NHS Foundation Trust	•University Hospital Southampton NHS Foundation Trust<br>•University Hospitals Birmingham NHS Foundation Trust<br>•University Hospitals Bristol And Weston NHS Foundation Trust<br>•University Hospitals Coventry And Warwickshire NHS Trust<br>•University Hospitals Dorset NHS Foundation Trust<br>•University Hospitals Of Derby And Burton NHS Foundation Trust<br>•University Hospitals Of Leicester NHS Trust<br>•University Hospitals Of Morecambe Bay NHS Foundation Trust<br>•University Hospitals Of North Midlands NHS Trust<br>•University Hospitals Plymouth NHS Trust<br>•University Hospitals Sussex NHS Foundation Trust<br>•Walsall Healthcare NHS Trust<br>•Warrington And Halton Teaching Hospitals NHS Foundation Trust<br>•West Hertfordshire Hospitals NHS Trust<br>•West Suffolk NHS Foundation Trust<br>•Whittington Health NHS Trust<br>•Wirral University Teaching Hospital NHS Foundation Trust<br>•Worcestershire Acute Hospitals NHS Trust<br>•Wrightington, Wigan And Leigh NHS Foundation Trust<br>•Wye Valley NHS Trust<br>•Yeovil District Hospital NHS Foundation Trust<br>•York And Scarborough Teaching Hospitals NHS Foundation Trust

Table S1. The names of the Hospital Trusts included in the national R2R reporting analysis
Legend. Data is presented anonymously in associated figures.

For peer review only

Procedure	OPCS Codes
Lower limb surgery within 48hrs	See Online supplement xls.
Thorax-abdominal-pelvic surgery with 72hrs	See Online supplement xls.
Invasive procedure within 24hrs	See Online supplement xls.

Table S2. Codes used to identify surgical interventions.
Legend. OPCS = OPCS Classification of Interventions and Procedures code used to identify the coded clinical entry.

	**2019**	**Remain**		
		Yes (+)	No (-)	Total
**eR2Ra**	Yes (+)	214,613	21,333	235,946
	No (-)	128,470	19,270	147,740
	Total	343,083	40,603	383,686
	**2020**	**Remain**		
		Yes (+)	No (-)	Total
**eR2Ra**	Yes (+)	177,852	18,283	196,135
	No (-)	97,119	17,606	114,725
	Total	274,971	35,889	310,860
	**2021**	**Remain**		
		Yes (+)	No (-)	Total
**eR2Ra**	Yes (+)	208,449	19,989	228,438
	No (-)	115,111	19,075	134,186
	Total	323,560	39,064	362,624

	**2019**	**Remain**		
		Yes (+)	No (-)	Total
**eR2Rab**	Yes (+)	301,018	30,176	331,194
	No (-)	42,065	10,427	52,492
	Total	343,083	40,603	383,686
	**2020**	**Remain**		
		Yes (+)	No (-)	Total
**eR2Rab**	Yes (+)	249,964	28,636	278,600
	No (-)	25,007	7,253	32,260
	Total	274,971	35,889	310,860
	**2021**	**Remain**		
		Yes (+)	No (-)	Total
**eR2Rab**	Yes (+)	291,576	30,847	322,423
	No (-)	31,984	8,217	40,201
	Total	323,560	39,064	362,624

Table S3a. Contingency tables showing the number of patients meeting criteria for eR2Ra and eR2ab and the corresponding number of patients who remain in hospital over the next 24 hours or do not (were discharged), for the in-patient population at 08.00.

For peer review only

	2019	Remain		
		Yes (+)	No (-)	Total
**eR2Ra**	Yes (+)	214,005	19,919	233,924
	No (-)	129,543	18,465	148,008
	Total	343,548	38,384	381,932
	**2020**	**Remain**		
		Yes (+)	No (-)	Total
**eR2Ra**	Yes (+)	178,709	17,343	196,052
	No (-)	98,123	17,692	115,815
	Total	276,832	35,035	311,867
	**2021**	**Remain**		
		Yes (+)	No (-)	Total
**eR2Ra**	Yes (+)	211,080	19,105	230,185
	No (-)	116,893	19,616	136,509
	Total	327,973	38,721	366,694

	2019	Remain		
		Yes (+)	No (-)	Total
**eR2Rab**	Yes (+)	299,551	28,334	327,885
	No (-)	43,997	10,050	54,047
	Total	343,548	38,384	381,932
	**2020**	**Remain**		
		Yes (+)	No (-)	Total
**eR2Rab**	Yes (+)	250,507	27,672	278,179
	No (-)	26,325	7,363	33,688
	Total	276,832	35,035	311,867
	**2021**	**Remain**		
		Yes (+)	No (-)	Total
**eR2Rab**	Yes (+)	294,260	30,038	324,298
	No (-)	33,713	8,683	42,396
	Total	327,973	38,721	366,694

Table S3b. Contingency tables showing the number of patients meeting criteria for eR2Ra and eR2ab and the corresponding number of patients who remain in hospital over the next 24 hours or do not (were discharged), for the in-patient population at 16.00.

	eR2Ra			eR2Rab		
Year	2019	2020	2021	2019	2020	2021
Sensitivity	0.63	0.65	0.65	0.88	0.91	0.90
Specificity	0.46	0.47	0.47	0.24	0.18	0.19
J statistic	9%	12%	12%	11%	9%	9%

Table S4. The Sensitivity, Specificity and J statistic calculations for Data presented in Table 5 of the main manuscript. A Contingency table showing the number of patients meeting criteria for eR2Ra and eR2ab

For peer review only

Population at 00:00	Not meeting eR2Rab criteria and discharged in subsequent 24 hours	Not meeting eR2Rab criteria and not discharged
n	22515	97326
**Age in years**: median (IQR)	60(45-74)	64(49-77)
**Sex (n, %)**	.	
Female	10833 (48.1%)	45345 (46.6%)
Male	11682 (51.9%)	51981 (53.4%)
**Self-reported ethnicity (n, %)**		
White	15761 (70.0%)	70194 (72.1%)
Mixed/ Multiple	411 (1.8%)	1549 (1.6%)
Asian/ Asian British	2952 (13.1%)	12194 (12.5%)
Black/ African/ Caribbean/ Black British	1274 (5.7%)	5857 (6.0%)
Other ethnic group	567 (2.5%)	2285 (2.3%)
Not known	1550 (6.9%)	5247 (5.4%)
**Co-morbidity count (n, %)**		
None	6544 (29.1%)	24873 (25.6%)
1-2	10321 (45.8%)	41401 (42.5%)
3 or more	5650 (25.1%)	31052 (31.9%)
**Morbidities (n, %)**		
Hypertension	9168 (40.7%)	43062 (44.2%)
Cerebrovascular disease	1512 (6.7%)	10128 (10.4%)
Atrial fibrillation	2947 (13.1%)	17096 (17.6%)
Ischaemic heart disease, angina, myocardial infarct	3810 (16.9%)	20962 (21.5%)
Diabetes (type 1 and 2)	4809 (21.4%)	24368 (25.0%)
Asthma	2644 (11.7%)	9899 (10.2%)
COPD	1594 (7.1%)	7255 (7.5%)
Interstitial Lung Disease	24 (0.1%)	129 (0.1%)
Chronic Kidney Disease	3135 (13.9%)	16633 (17.1%)
Any active Malignancy	3968 (17.6%)	17572 (18.1%)
Dementia (all types)	535 (2.4%)	3413 (3.5%)
**English Indices of deprivation**		
1	9448 (42.0%)	38534 (39.6%)
2	4638 (20.6%)	19841 (20.4%)
3	3888 (17.3%)	16630 (17.1%)
4	2200 (9.8%)	9432 (9.7%)
5	1644 (7.3%)	6914 (7.1%)
Missing	697 (3.1%)	5975 (6.1%)
Regained R2R criteria during stay? (n, %)	N/A	58609 (60.2%)
**Reason for regaining R2R criteria?**	N/A	
ICU		1727 (1.8%)
TAP surgery (72h)		263 (0.3%)
Lower limb surgery (24h)		95 (0.1%)
Invasive surgery (24h)		579 (0.6%)
Acute dependency level (48h)		7452 (7.7%)
NEWS >3 (24h)		20605 (21.2%)
O2 treatment (24h)		5111 (5.3%)

For peer review only

Intravenous fluids or treatments (24 hours, > tds)		27069 (27.8%)
GCS < or + 12 (24h)		183 (0.2%)
EOL care (24h)		165 (0.2%)
Increased dependency (48h)		10290 (10.6%)

Table S5. Demographics of patients not meeting R2R criteria on presentation to QEHB in the censor period. Legend. Data is number (percentage) of patients in a bed at 00:00 who either were or were not discharged in the subsequent twenty-four hours after eR2R assessment. Ethnicity was self-reported. Medical conditions were physician confirmed and checked against admission and linked primary care notes. English Indices of deprivation were calculated using postcode.

For peer review only

Online Supplement Figures

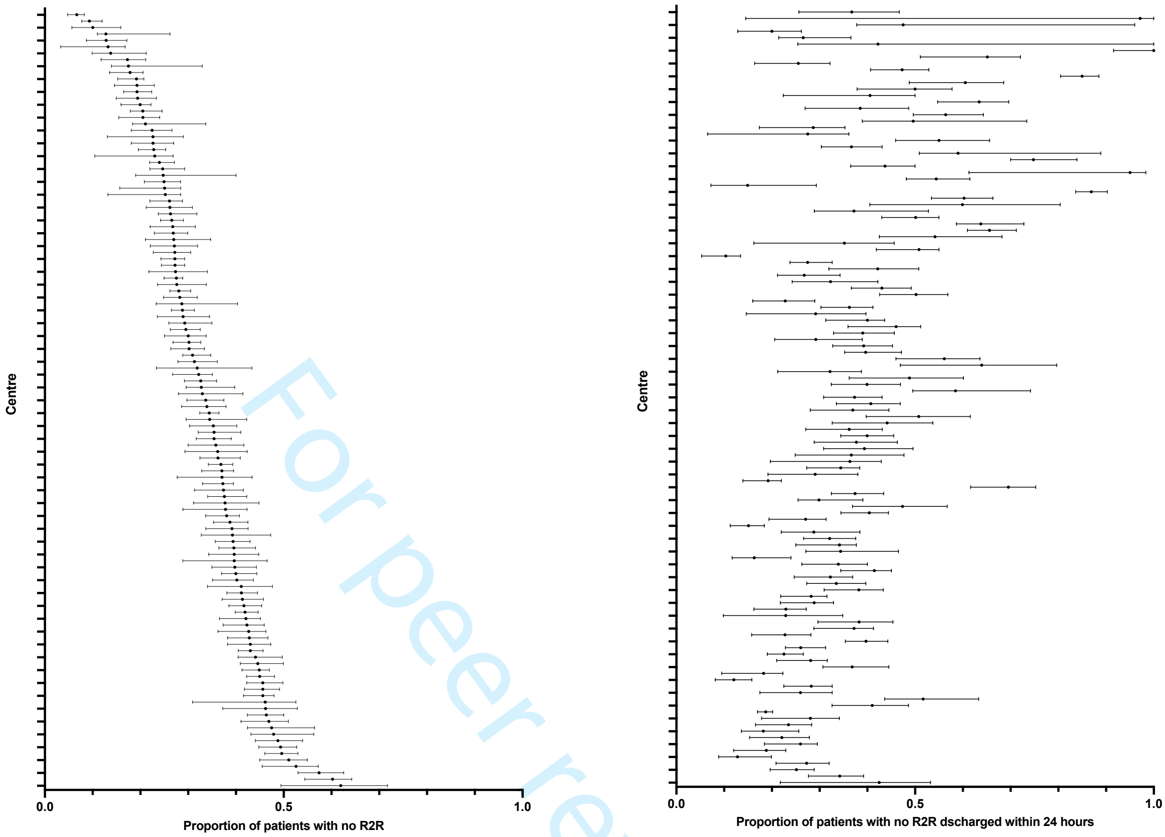

Figure S1. The proportions of patients with no right to reside (A), and proportions of these with no right to reside that were discharged within 24 hours (B). Analysis by week.

Legend: The proportions of patients not meeting the R2R, and the proportions of these patients that were discharged within 24 hours were extracted from daily reports for each national NHS centre. Data is presented as the median and IQR for the reporting period. We have ordered centres in both Figure 1 a and 1 b according to the median value of proportion of patients with R2R.

For peer review only

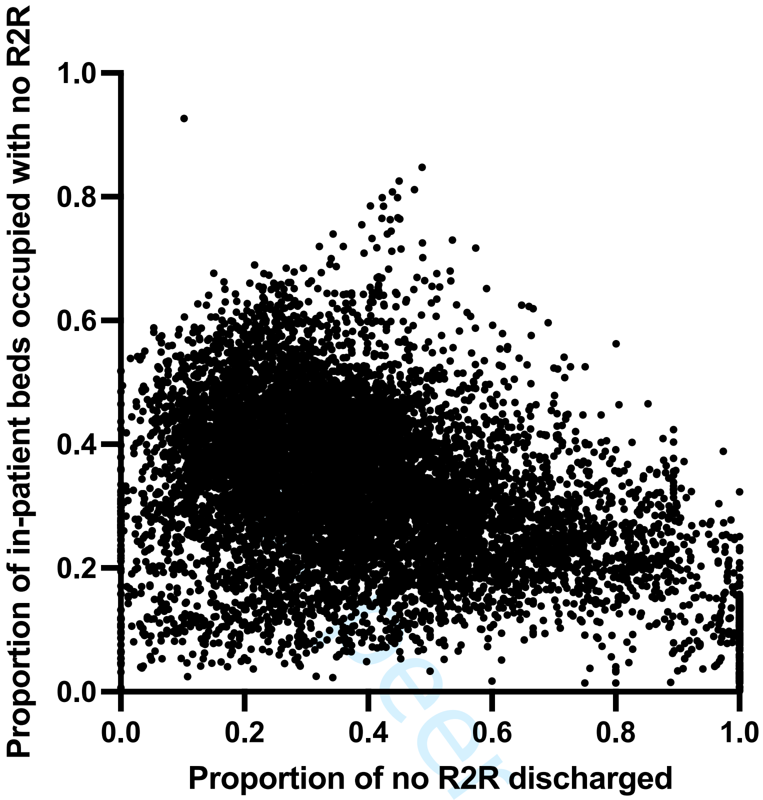

Figure S2. The proportions of patients with no R2R and of that group the proportion discharged over the next 24 hours

Legend: The proportions of patients not meeting the R2R, and of that group the proportion of patients discharged within 24 hours, reported to SDCS from 29 Nov 2021 – 20 Feb 2022 across 121 centres. Each dot represents result for a single centre-day. The two metrics were associated (slope = -0.21, $p<0.0001$) but the correlation was low (R^2=0.12).

For peer review only

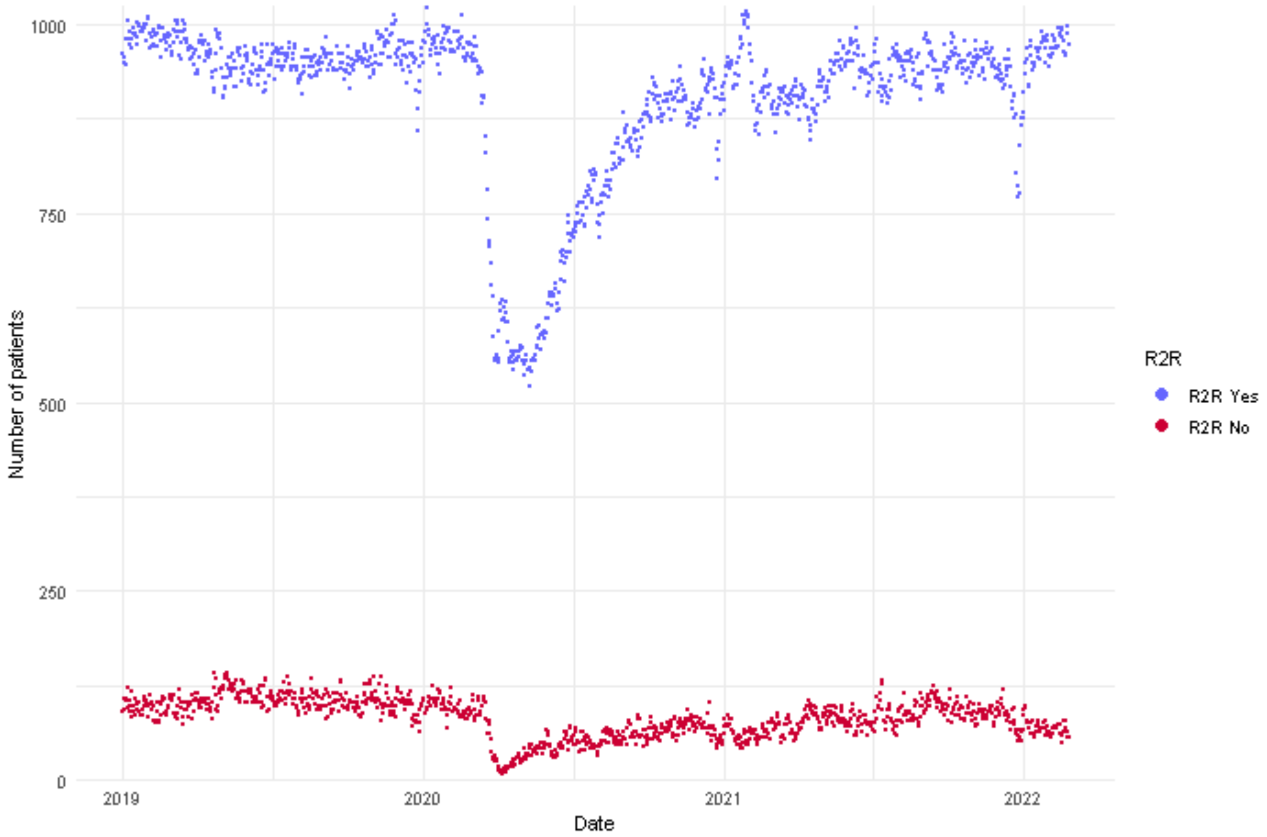

Figure S3. The number of patients meeting or not meeting eR2Rab criteria 01 Jan 2019 - 31 Dec 2021

Legend: Number of patients with (red dot) or without (blue dot) eR2Rab at 00:00 on each day of 2019-2021. The first COVID-19 admission to QEHB occurred on 1st March 2020. The first wave of the pandemic was associated with significant changes resulting in reduced bed occupancy and the majority of admitted patients had a diagnosis of COVID-19

For peer review only

STROBE Statement—checklist of items that should be included in reports of observational studies

	Item No	Recommendation	Page
**Title and abstract**	1	(*a*) Indicate the study's design with a commonly used term in the title or the abstract	1
		(*b*) Provide in the abstract an informative and balanced summary of what was done and what was found	2
**Introduction**			
Background/rationale	2	Explain the scientific background and rationale for the investigation being reported	3
Objectives	3	State specific objectives, including any prespecified hypotheses	4
**Methods**			
Study design	4	Present key elements of study design early in the paper	6
Setting	5	Describe the setting, locations, and relevant dates, including periods of recruitment, exposure, follow-up, and data collection.	6
Participants	6	(*a*) *Cohort study*—Give the eligibility criteria, and the sources and methods of selection of participants. Describe methods of follow-up<br>*Case-control study*—Give the eligibility criteria, and the sources and methods of case ascertainment and control selection. Give the rationale for the choice of cases and controls<br>*Cross-sectional study*—Give the eligibility criteria, and the sources and methods of selection of participants	6
Variables	7	Clearly define all outcomes, exposures, predictors, potential confounders, and effect modifiers. Give diagnostic criteria, if applicable	6
Data sources/ measurement	8*	For each variable of interest, give sources of data and details of methods of assessment (measurement). Describe comparability of assessment methods if there is more than one group	6
Bias	9	Describe any efforts to address potential sources of bias	6
Study size	10	Explain how the study size was arrived at	6
Quantitative variables	11	Explain how quantitative variables were handled in the analyses.	6
Statistical methods	12	(*a*) Describe all statistical methods, including those used to control for confounding	6
		(*b*) Describe any methods used to examine subgroups and interactions	6
		(*c*) Explain how missing data were addressed	6
		(*d*) *Cohort study*—If applicable, explain how loss to follow-up was addressed<br>*Case-control study*—If applicable, explain how matching of cases and controls was addressed<br>*Cross-sectional study*—If applicable, describe analytical methods taking account of sampling strategy	6

For peer review only - http://bmjopen.bmj.com/site/about/guidelines.xhtml

**Results**			
Participants	13*	(a) Report numbers of individuals at each stage of study—eg numbers potentially eligible, examined for eligibility, confirmed eligible, included in the study, completing follow-up, and analysed.	7
		(b) Give reasons for non-participation at each stage	7
		(c) Consider use of a flow diagram **Online supplement**	
Descriptive data	14*	(a) Give characteristics of study participants (eg demographic, clinical, social) and information on exposures and potential confounders.	9
		(b) Indicate number of participants with missing data for each variable of interest	9
		(c) *Cohort study*—Summarise follow-up time (eg, average and total amount). N/A	
Outcome data	15*	*Cohort study*—Report numbers of outcome events or summary measures over time. N/A	
		Case-control study—Report numbers in each exposure category, or summary measures of exposure. N/A	8-10
		Cross-sectional study—Report numbers of outcome events or summary measures	
Main results	16	(*a*) Give unadjusted estimates and, if applicable, confounder-adjusted estimates and their precision (eg, 95% confidence interval). Make clear which confounders were adjusted for and why they were included	8-12
		(*b*) Report category boundaries when continuous variables were categorized	8-12
		(*c*) If relevant, consider translating estimates of relative risk into absolute risk for a meaningful time period.	8-12
Other analyses	17	Report other analyses done—eg analyses of subgroups and interactions, and sensitivity analyses	8-12
**Discussion**			
Key results	18	Summarise key results with reference to study objectives.	13-14
Limitations	19	Discuss limitations of the study, sources of potential bias or imprecision. Discuss both direction and magnitude of any potential bias	13-14
Interpretation	20	Give a cautious overall interpretation of results considering objectives, limitations, multiplicity of analyses, results from similar studies, and other relevant evidence. 13-14	
Generalisability	21	Discuss the generalisability (external validity) of the study results 14	
**Other information**			
Funding	22	Give the source of funding and the role of the funders for the present study and, if applicable, for the original study on which the present article is based. 15	

For peer review only
